# Supplementary material for: An Exploratory Analysis of Essential Tremor and Associated Phenotypes
Source: Tremor Other Hyperkinet Mov (N Y). 2026 Apr 28;16:27. doi: 10.5334/tohm.1168 (PMC13131336; doi:10.5334/tohm.1168)
Supplement: Supplementary Material. — Supplementary Files and Sensitivity Analyses. [file tohm-16-1-1168-s1.pdf]

Supplemental Figures

Sensitivity Analyses

Sensitivity analyses done for all Mendelian Randomization (MR) analyses are shown below.

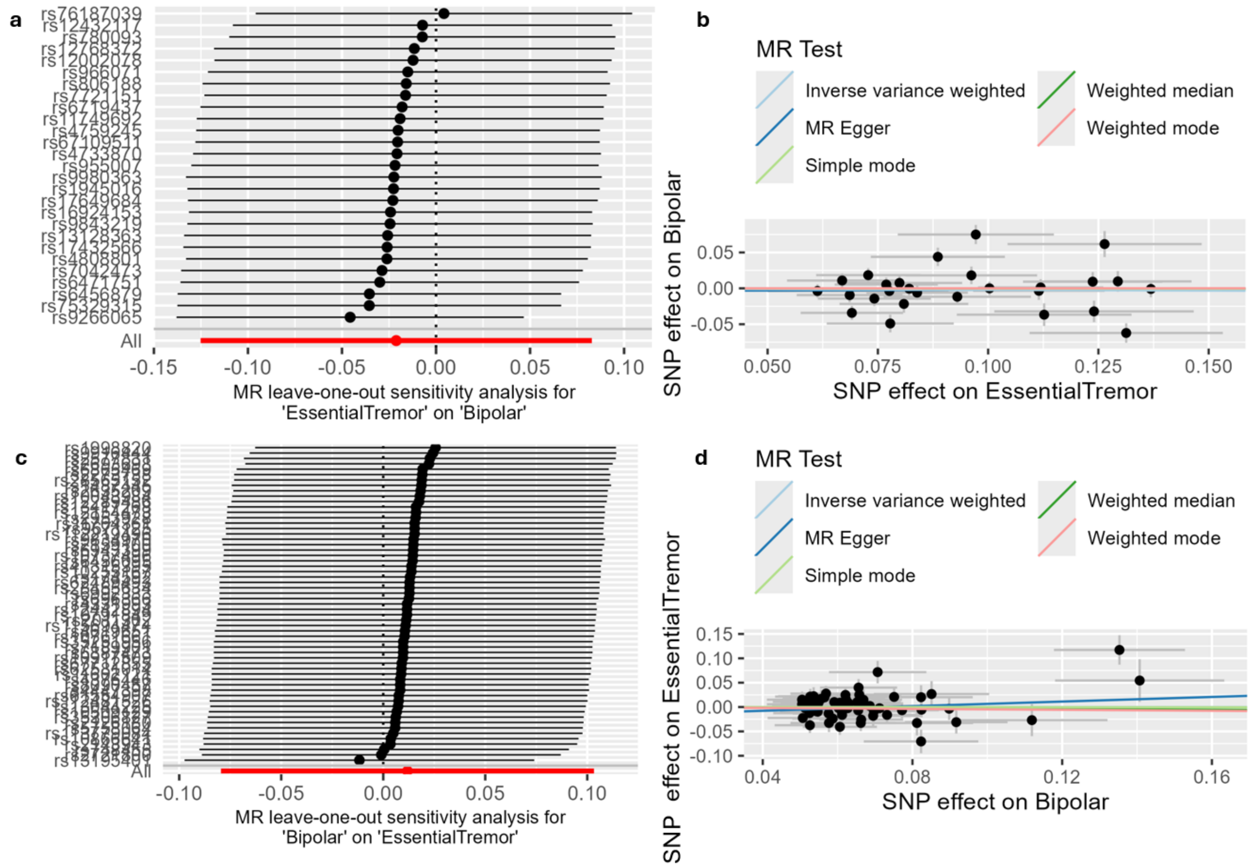

Figure S1: Bipolar and ET MR sensitivity analyses. Sensitivity analyses for (A) leave-one-out analysis of ET on bipolar disorder and (B) SNP effect MR test for when ET is the exposure and bipolar disorder is the outcome are shown. Sensitivity analyses for (C) leave-one-out analysis of bipolar disorder on ET and (D) SNP effect MR test for when bipolar is the exposure and ET is the outcome are also shown.

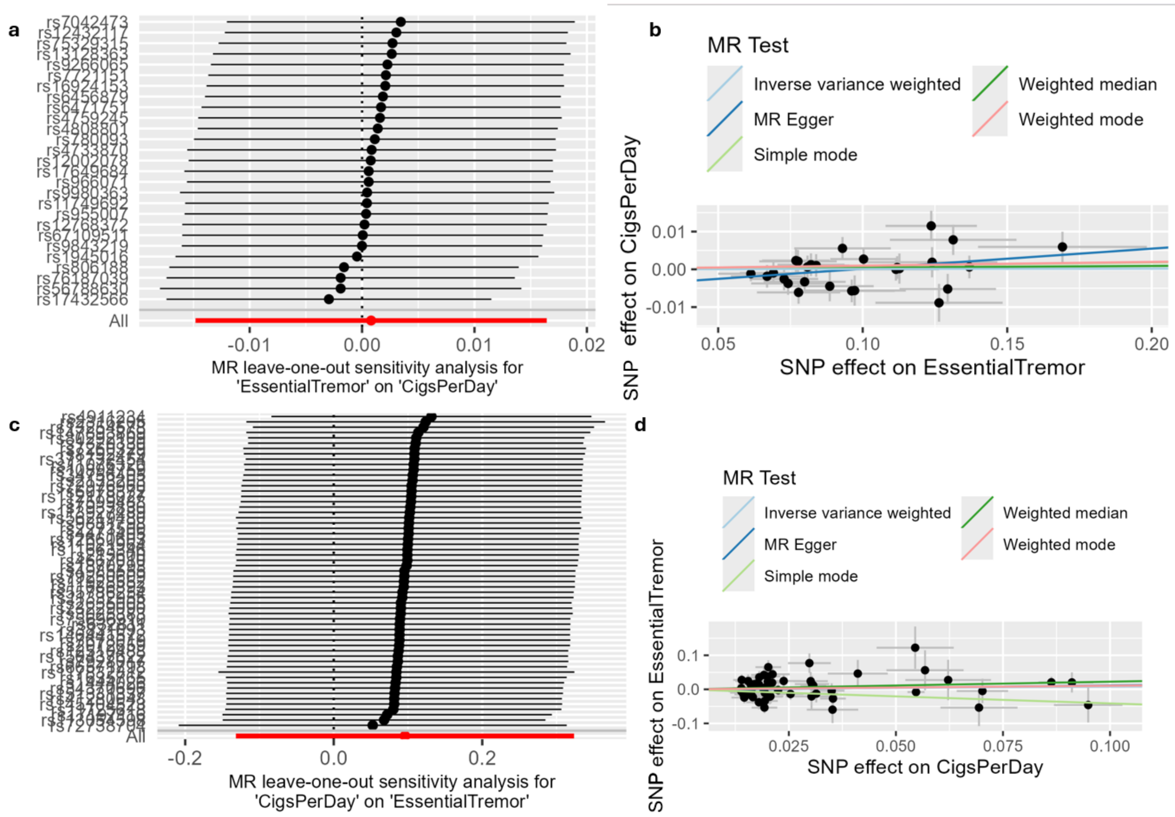

Figure S2: Smoking and ET MR sensitivity analyses. Sensitivity analyses for (A) leave-one-out analysis of ET on smoking and (B) SNP effect MR test for when ET is the exposure and smoking is the outcome are shown. Sensitivity analyses for (C) leave-one-out analysis of smoking on ET and (D) SNP effect MR test for when smoking is the exposure and ET is the outcome are also shown.

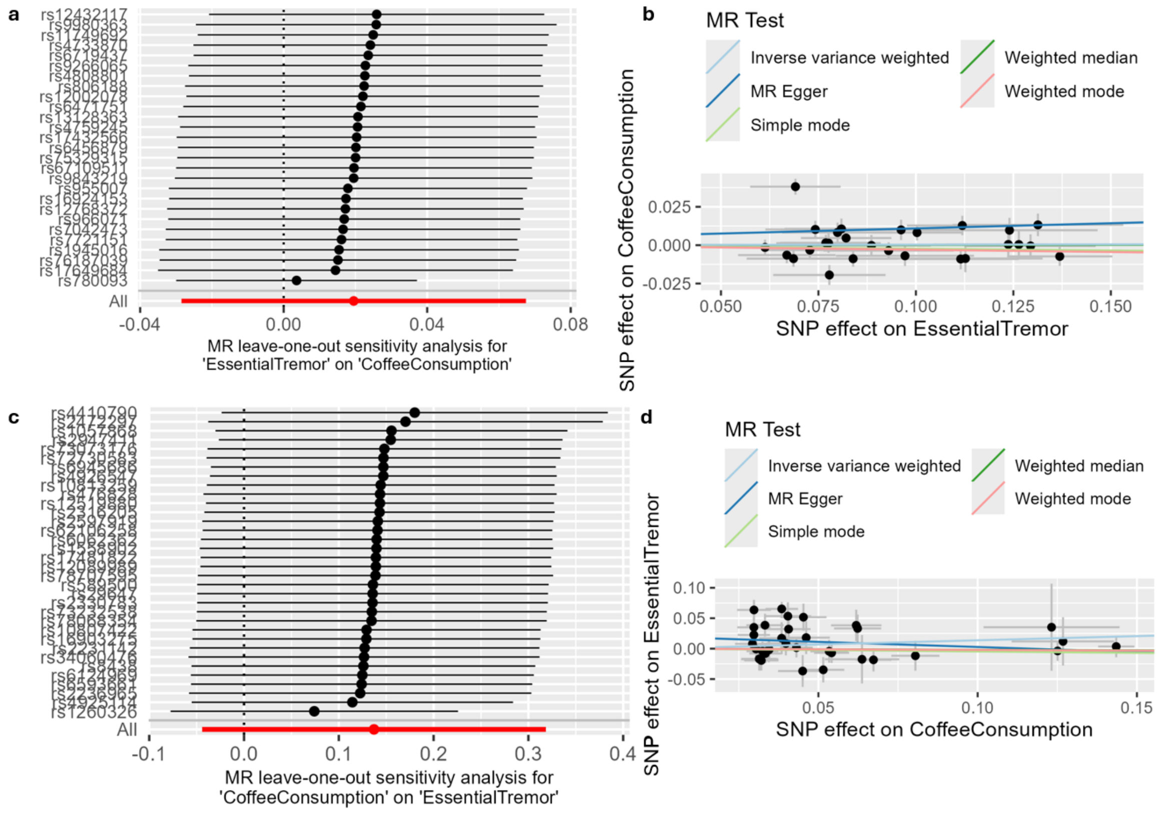

Figure S3: Coffee Consumption and ET MR sensitivity analyses. Sensitivity analyses for (A) leave-one-out analysis of ET on coffee consumption and (B) SNP effect MR test for when ET is the exposure and coffee consumption is the outcome are shown. Sensitivity analyses for (C) leave-one-out analysis of coffee consumption on ET and (D) SNP effect MR test for when coffee consumption is the exposure and ET is the outcome are also shown.

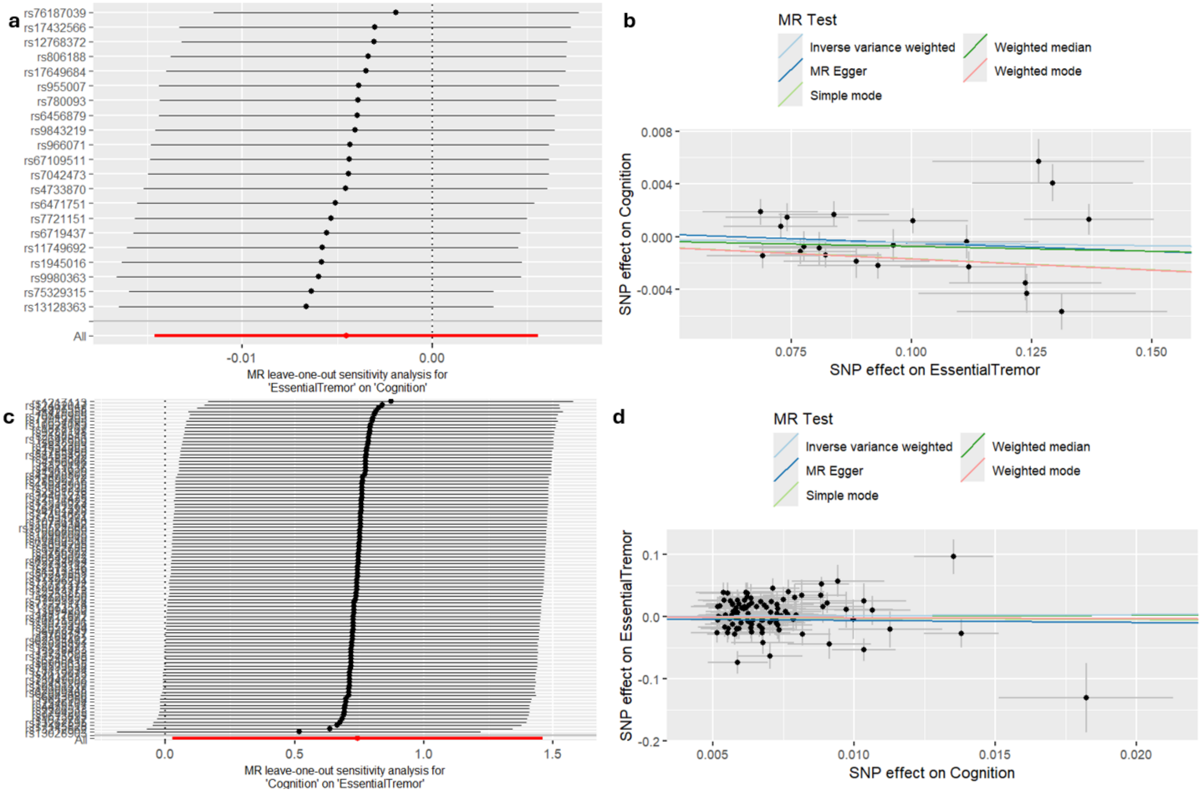

Figure S4: Cognition and ET MR sensitivity analyses. Sensitivity analyses for (A) leave-one-out analysis of ET on cognition and (B) SNP effect MR test for when ET is the exposure and cognition is the outcome are shown. Sensitivity analyses for (C) leave-one-out analysis of cognition on ET and (D) SNP effect MR test for when cognition is the exposure and ET is the outcome are also shown.

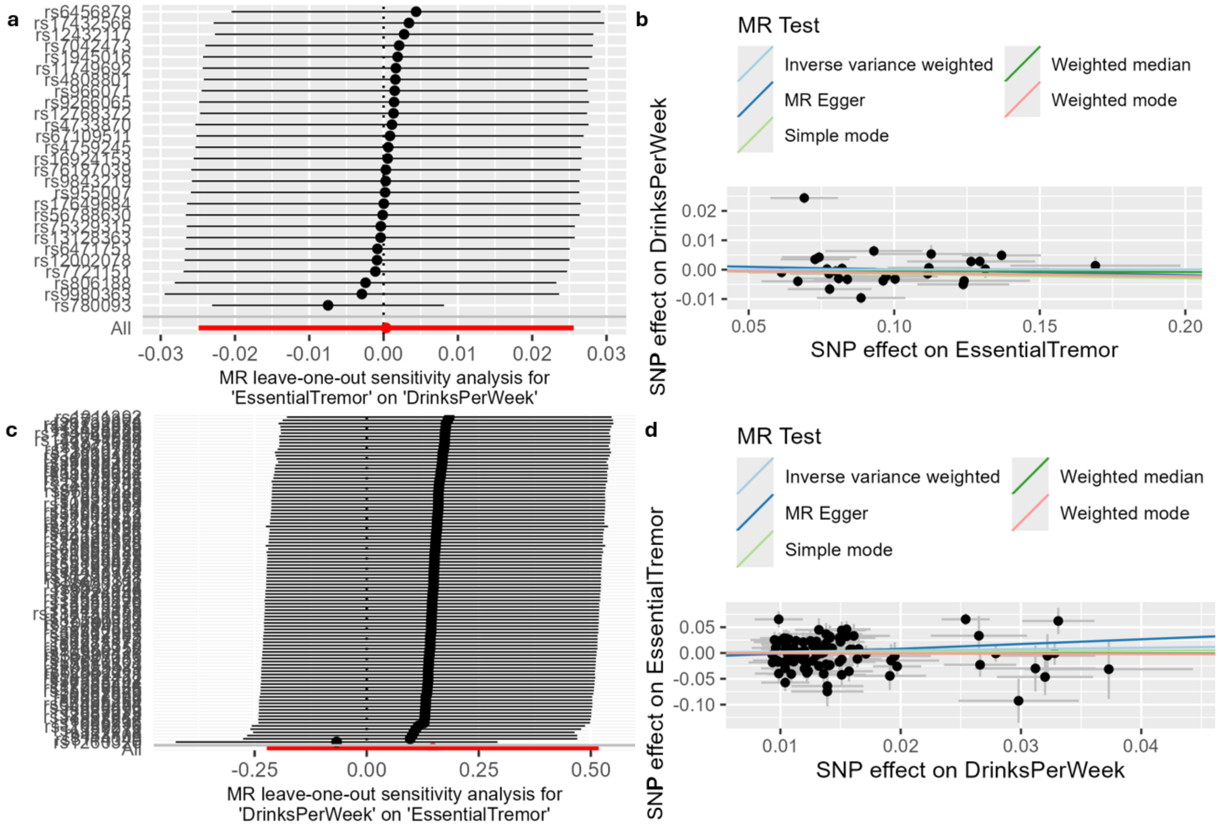

Figure S5: Alcohol intake and ET MR sensitivity analyses. Sensitivity analyses for (A) leave-one-out analysis of ET on alcohol intake and (B) SNP effect MR test for when ET is the exposure and alcohol intake is the outcome are shown. Sensitivity analyses for (C) leave-one-out analysis of alcohol intake on ET and (D) SNP effect MR test for when alcohol intake is the exposure and ET is the outcome are also shown.

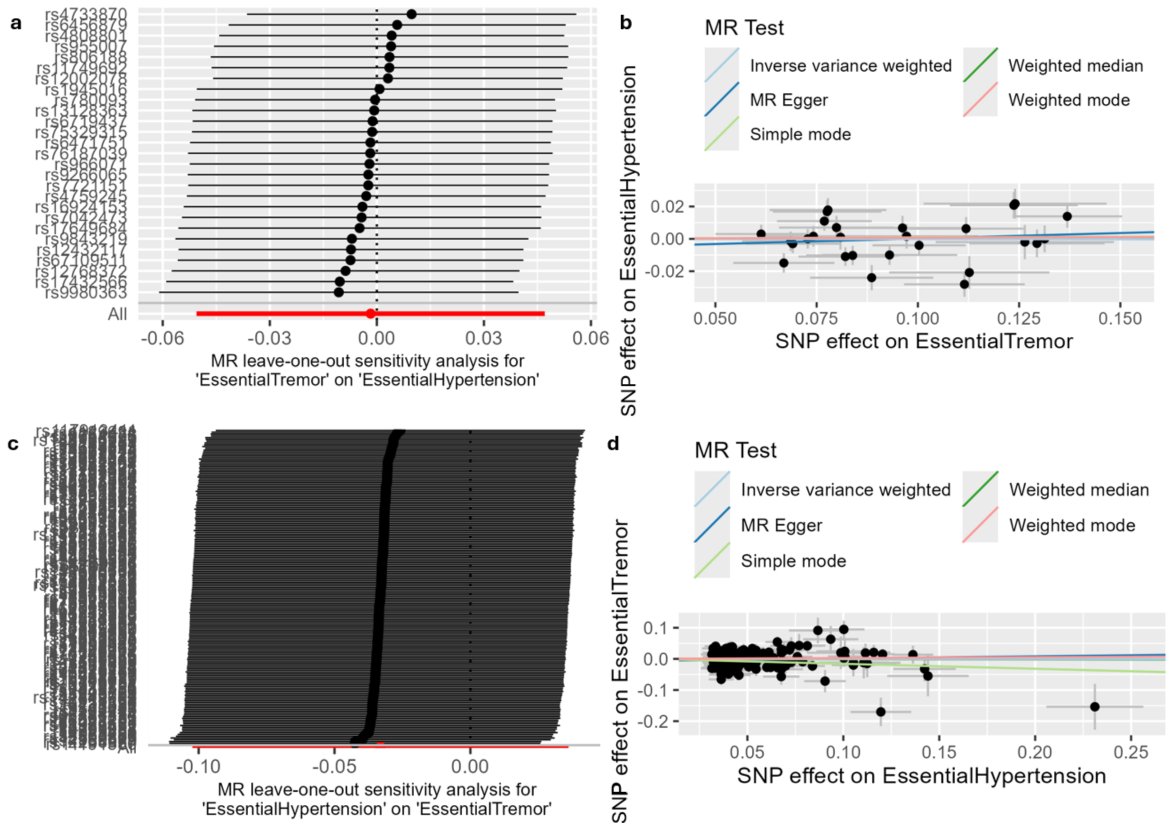

Figure S6: Essential hypertension and ET MR sensitivity analyses. Sensitivity analyses for (A) leave-one-out analysis of ET on essential hypertension and (B) SNP effect MR test for when ET is the exposure and essential hypertension is the outcome are shown. Sensitivity analyses for (C) leave-one-out analysis of essential hypertension on ET and (D) SNP effect MR test for when essential hypertension is the exposure and ET is the outcome are also shown.

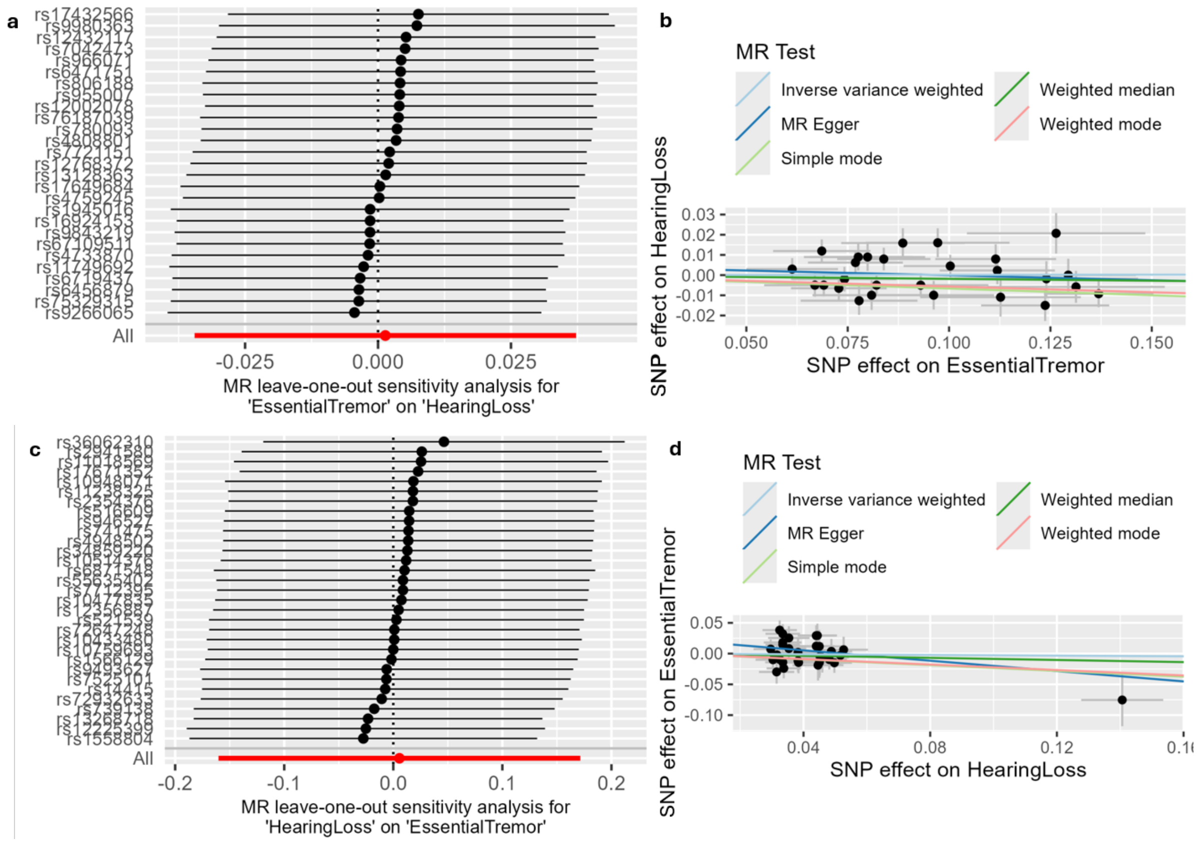

Figure S7: Hearing loss and ET MR sensitivity analyses. Sensitivity analyses for (A) leave-one-out analysis of ET on hearing loss and (B) SNP effect MR test for when ET is the exposure and hearing loss is the outcome are shown. Sensitivity analyses for (C) leave-one-out analysis of hearing loss on ET and (D) SNP effect MR test for when hearing loss is the exposure and ET is the outcome are also shown.

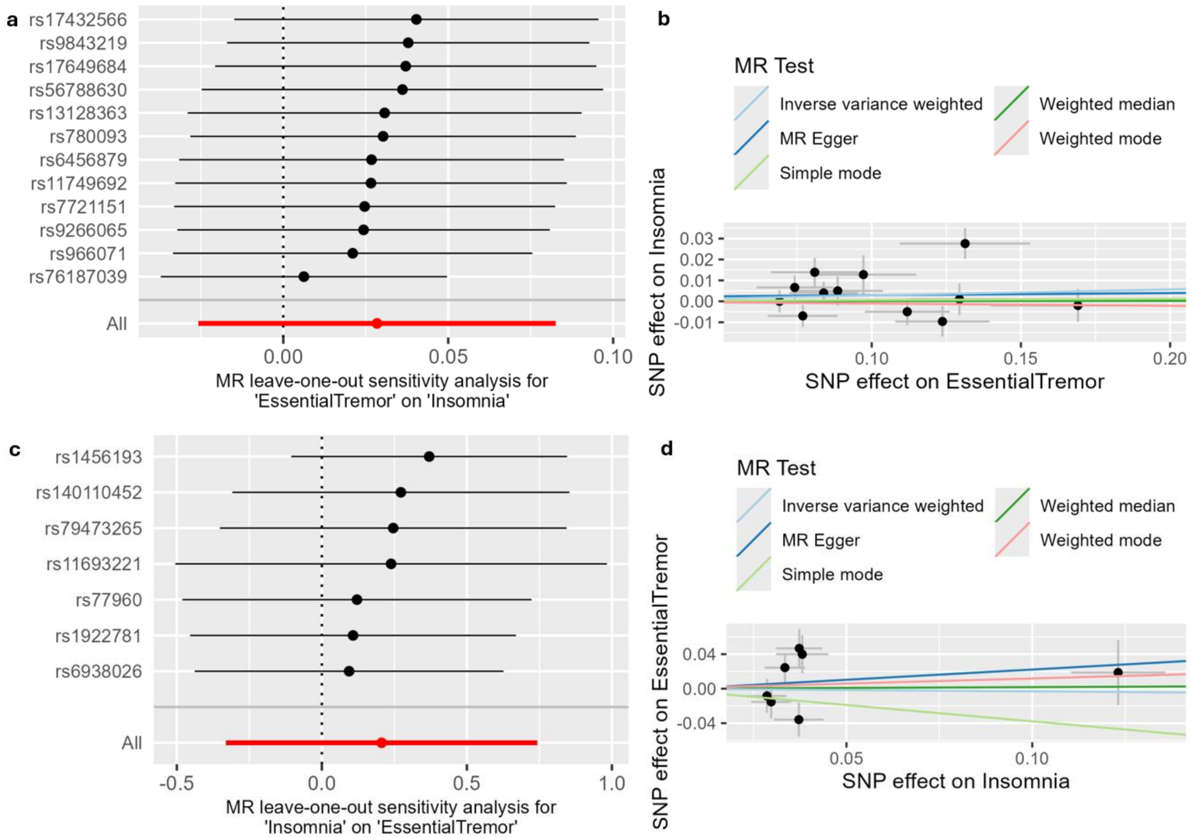

Figure S8: Insomnia and ET MR sensitivity analyses. Sensitivity analyses for (A) leave-one-out analysis of ET on insomnia and (B) SNP effect MR test for when ET is the exposure and insomnia is the outcome are shown. Sensitivity analyses for (C) leave-one-out analysis of insomnia on ET and (D) SNP effect MR test for when insomnia is the exposure and ET is the outcome are also shown.

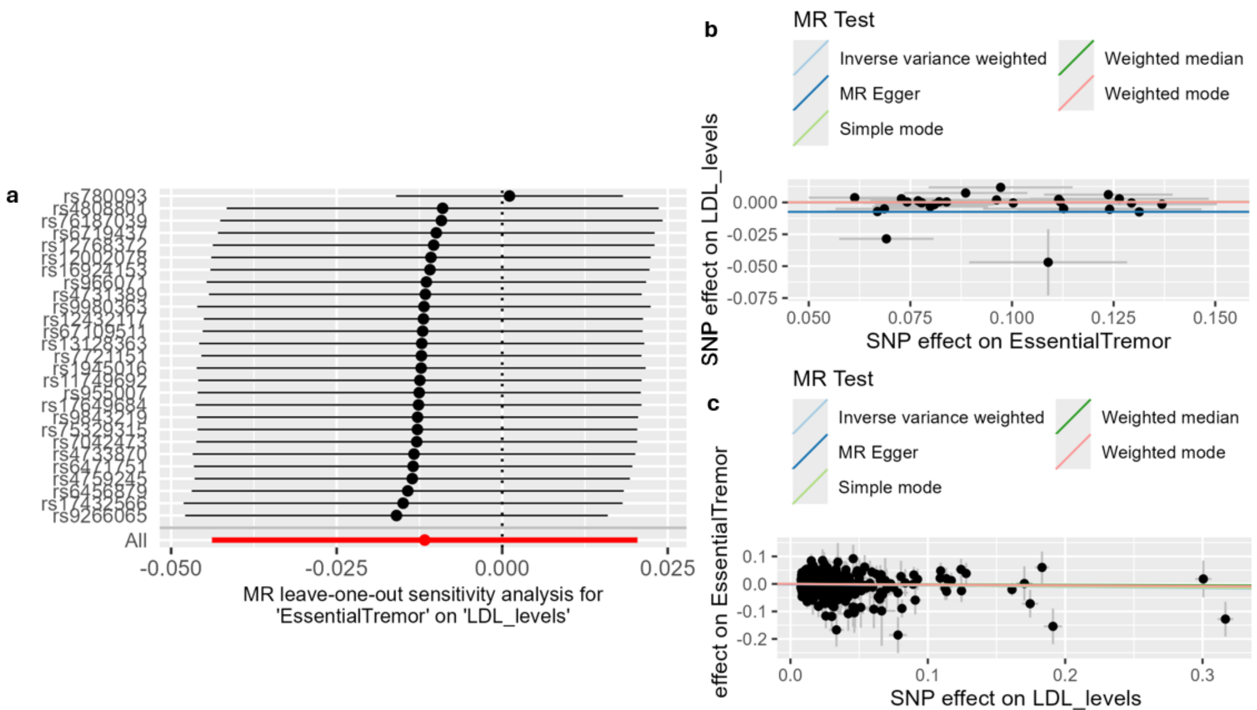

Figure S9: LDL levels and ET MR sensitivity analyses. Sensitivity analyses for (A) leave-one-out analysis of ET on LDL levels and (B) SNP effect MR test for when ET is the exposure and LDL levels is the outcome are shown. Sensitivity analyses for (C) leave-one-out analysis of LDL levels on ET and (D) SNP effect MR test for when LDL levels is the exposure and ET is the outcome are also shown.

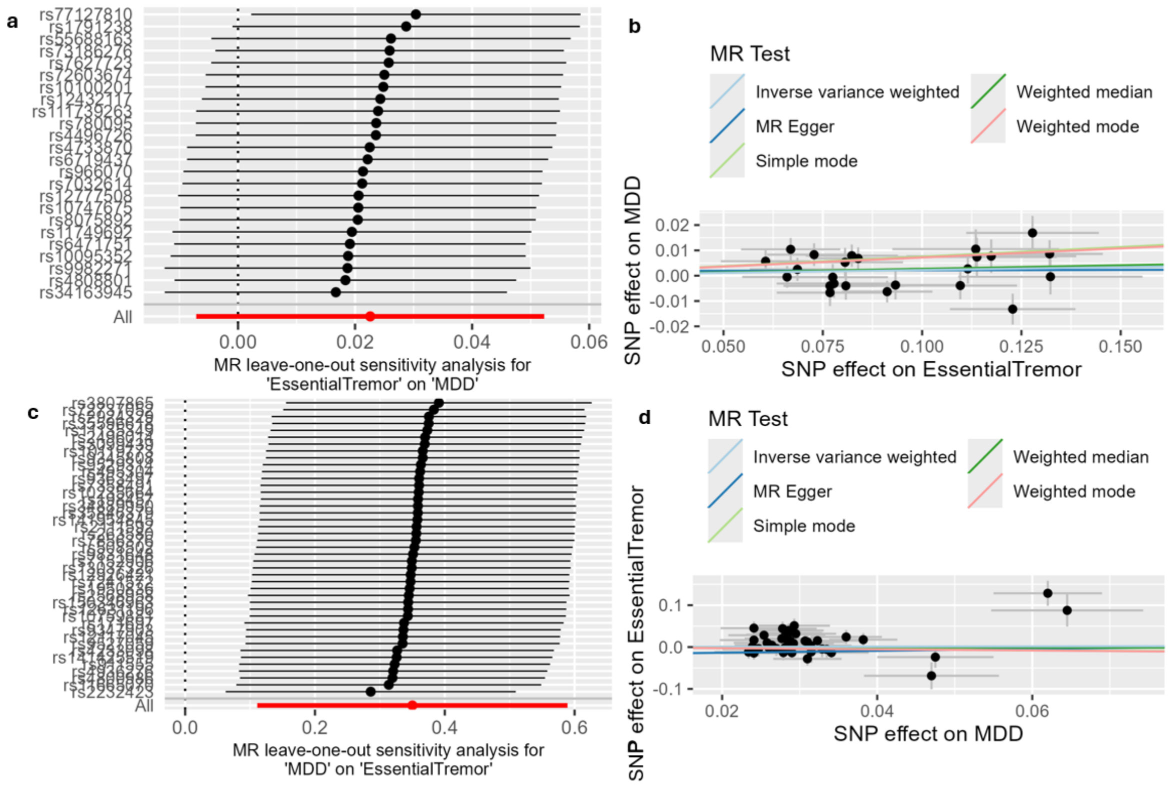

Figure S10: MDD and ET MR sensitivity analyses. Sensitivity analyses for (A) leave-one-out analysis of ET on MDD and (B) SNP effect MR test for when ET is the exposure and MDD is the outcome are shown. Sensitivity analyses for (C) leave-one-out analysis of MDD on ET and (D) SNP effect MR test for when MDD is the exposure and ET is the outcome are also shown.

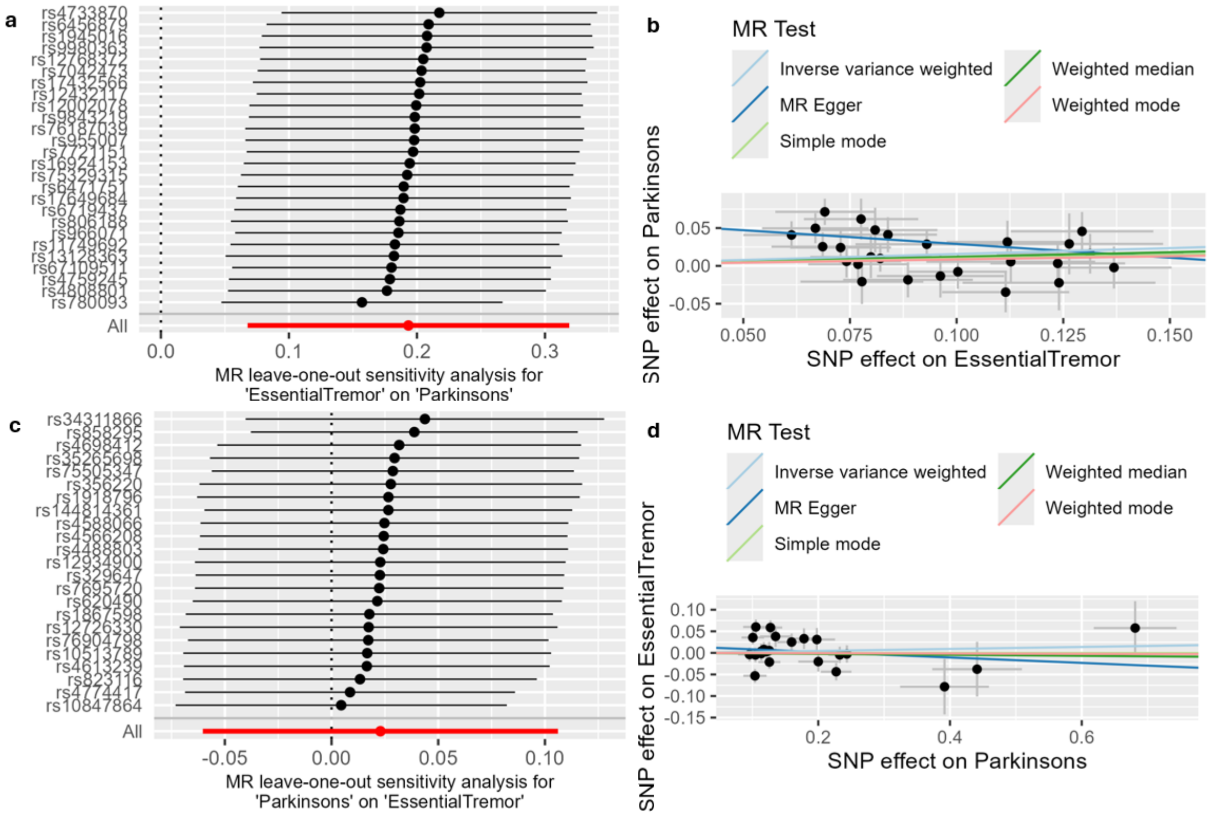

Figure S11: Parkinson's Disease and ET MR sensitivity analyses. Sensitivity analyses for (A) leave-one-out analysis of ET on Parkinson's disease and (B) SNP effect MR test for when ET is the exposure and Parkinson's disease is the outcome are shown. Sensitivity analyses for (C) leave-one-out analysis of Parkinson's disease on ET and (D) SNP effect MR test for when Parkinson's disease is the exposure and ET is the outcome are also shown.

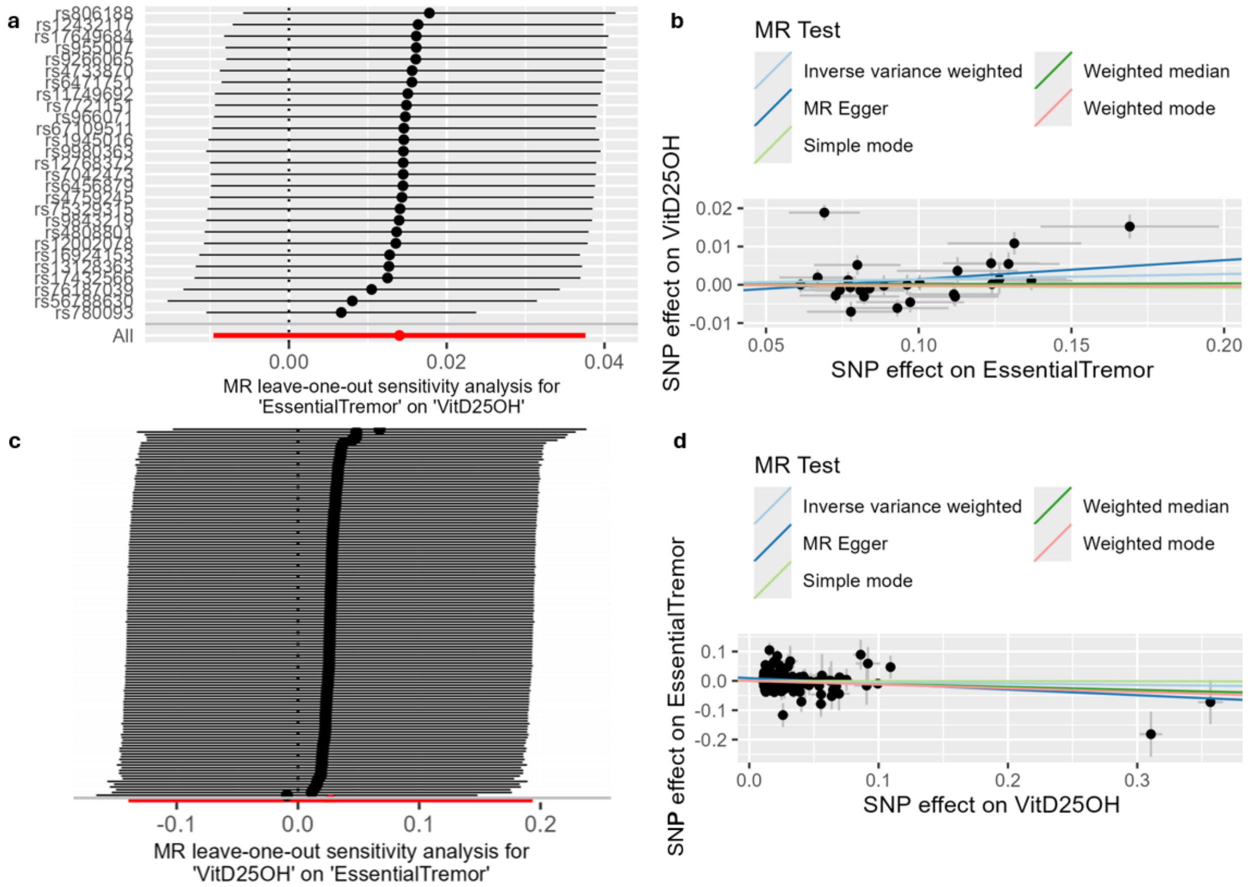

Figure S12: 25-OH Vit. D levels and ET MR sensitivity analyses. Sensitivity analyses for (A) leave-one-out analysis of ET on 25-OH Vit. D levels and (B) SNP effect MR test for when ET is the exposure and 25-OH Vit. D levels is the outcome are shown. Sensitivity analyses for (C) leave-one-out analysis of 25-OH Vit. D levels on ET and (D) SNP effect MR test for when 25-OH Vit. D levels is the exposure and ET is the outcome are also shown.

Mendelian Randomization Results

Below the results of the bidirectional Mendelian Randomization figures along with text descriptions of details on each analysis are presented.

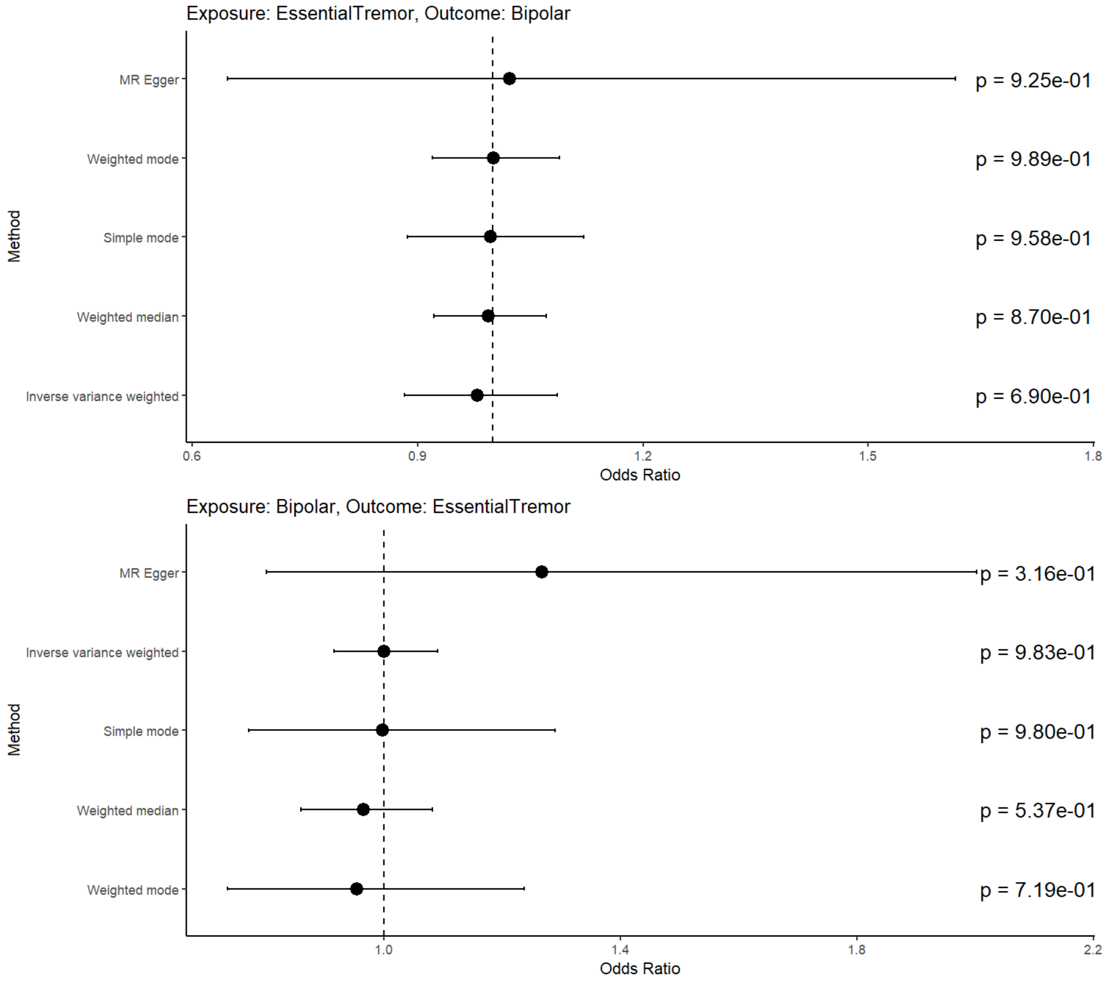

Figure S13: Bidirectional MR results for bipolar disorder and essential tremor. The forest plots depict MR analyses results when (A) essential tremor is the exposure with bipolar disorder as an outcome, and MR results when (B) bipolar disorder is the exposure and essential tremor is the outcome.

27 IVs were retained after clumping for the analysis with bipolar disorder as an outcome of ET. P-values were all far greater than the threshold p-value of 0.0016 after Bonferroni correction (**figure S13**). The global F-statistic for this analysis was 42.817, indicating that results are not driven by weak IVs. The PRESSO results detected some outlier SNPs, however the IVW result with these SNPs removed was still insignificant, with a p-value of 0.477. Odds ratios for the analysis were very similar (0.978 for PRESSO analysis, 0.979 for raw IVW results). This suggests that outlier SNPs are not significantly driving the results. No IVs were removed from Steiger

filtering. From the Leave-One-Out (LOO) plot (shown in **figure S1a**), no SNP seems to significantly skew the results towards the null hypothesis. The small y-intercept in the Egger regression (seen in **figure S1b**) suggests horizontal pleiotropy is minimally contributing to these results. For the reverse-direction (ET as an outcome of bipolar disorder), 58 IVs were retained. Similar to the forward-direction, no results were significant (**figure S13**). The global F-statistic for the analysis was 38.137, so weak instrument bias is not likely to drive this effect. The PRESSO outlier-removed analysis reduced the p-value from 0.983 to 0.546, however this remains insignificant. Only one instrument variable was removed due to Steiger filtering. This, combined with the small Egger y-intercept (seen in **figure S1d**), suggests that horizontal pleiotropy was not a large contributor to the results. The LOO plot shows that no single SNP significantly skewed results (**figure S1c**).

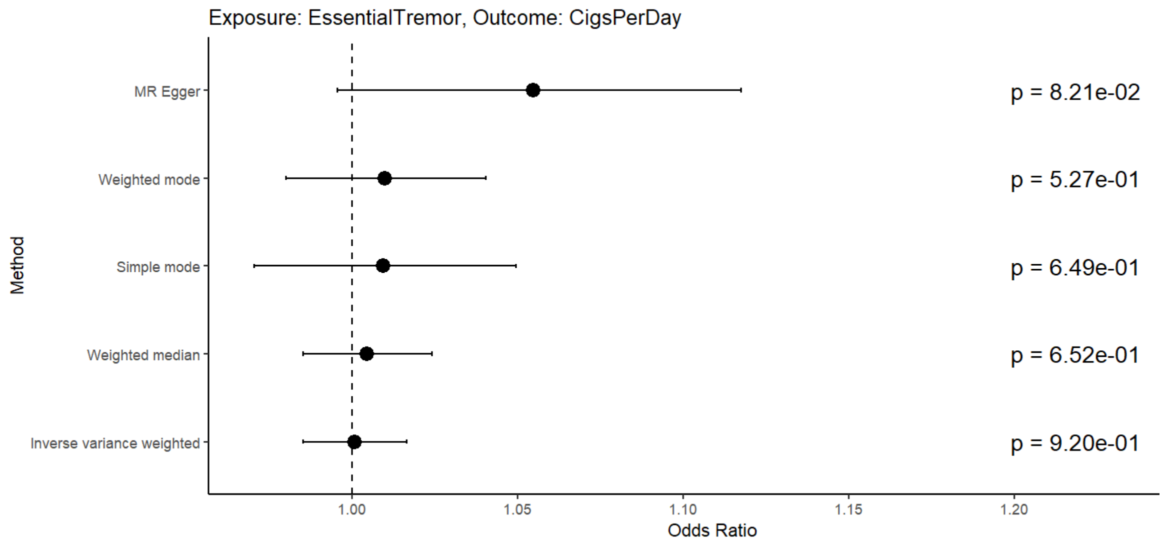

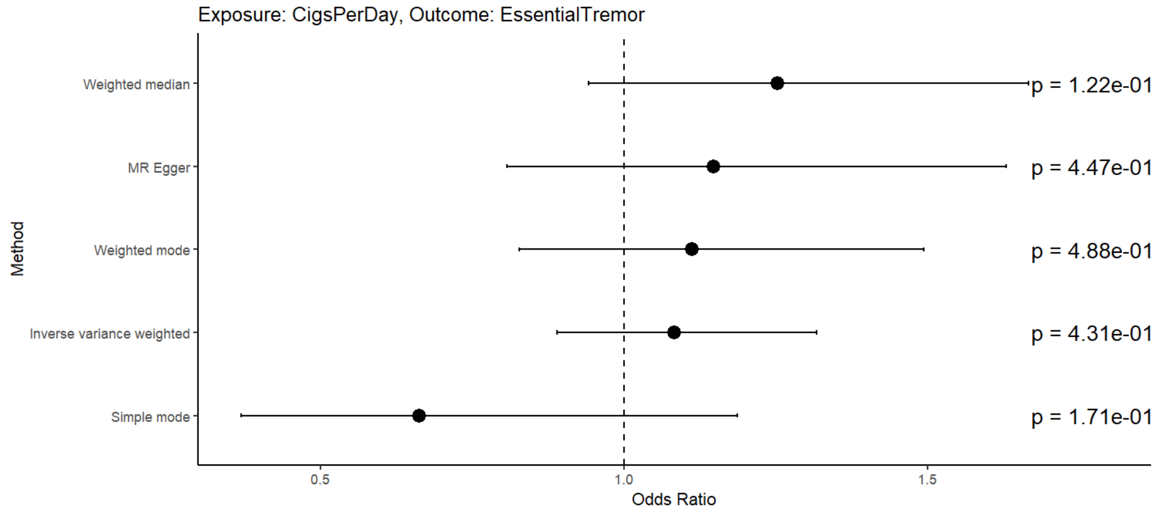

Figure S14: Bidirectional MR results for smoking and essential tremor. The forest plots depict MR analyses results when (A) essential tremor is the exposure with cigarettes smoked per day as an outcome, and MR results when (B) cigarettes smoked per day is the exposure and essential tremor is the outcome.

For ET as an exposure to cigarettes smoked per day, 27 IVs were retained for analysis. Again, no analytic method showed significance, as is seen in **figure S12**. The global F-statistic was 42.923, suggesting weak instrument bias was minimal. PRESSO failed to detect outliers, as did Steiger filtering. However, the Egger intercept (**figure S2b**) was notably larger than for other analytic methods, suggesting horizontal pleiotropy may be present. The LOO plot (**figure S2a**) shows that no IV seems to be singlehandedly driving the effect. For the reverse analysis, 56 IVs were retained after clumping, harmonizing and Steiger filtering. Most methods, apart from the simple mode, point towards a positive effect from the number of cigarettes smoked per day on ET, however results were not significant. The global F-statistic of 81.390 confirms that weak instrument bias is unlikely to be present. The LOO plot (**figure S2c**) seems to suggest that IV rs113054598 might be driving the association seen (β-effect estimate of 0.0678 with IV removed vs. 0.0959 with all IVs present), however, as the results point to a null association regardless, removing this SNP would not change the overall result. The Egger intercept is similar to that of other analytic methods, which are all very near 0, thus suggesting the method was unable to detect horizontal pleiotropy (**figure S2d**). PRESSO did not detect any outliers, but 3 IVs were removed during Steiger-filtering. As **figure S2d** shows, most IVs have a weak magnitude of effect on cigarettes per day, despite having a strong significance (as they were selected for having a p-value of less than 0.5×10^{-8} in the cigarettes per day GWAS). This could contribute to the result observed by the simple mode analysis, which does not weigh contributions to the global effect by IV exposure effect variance, contrary to other methods. Variance in the explanation of cigarettes smoked per day is therefore likely higher in those IVs suggesting a protective effect.

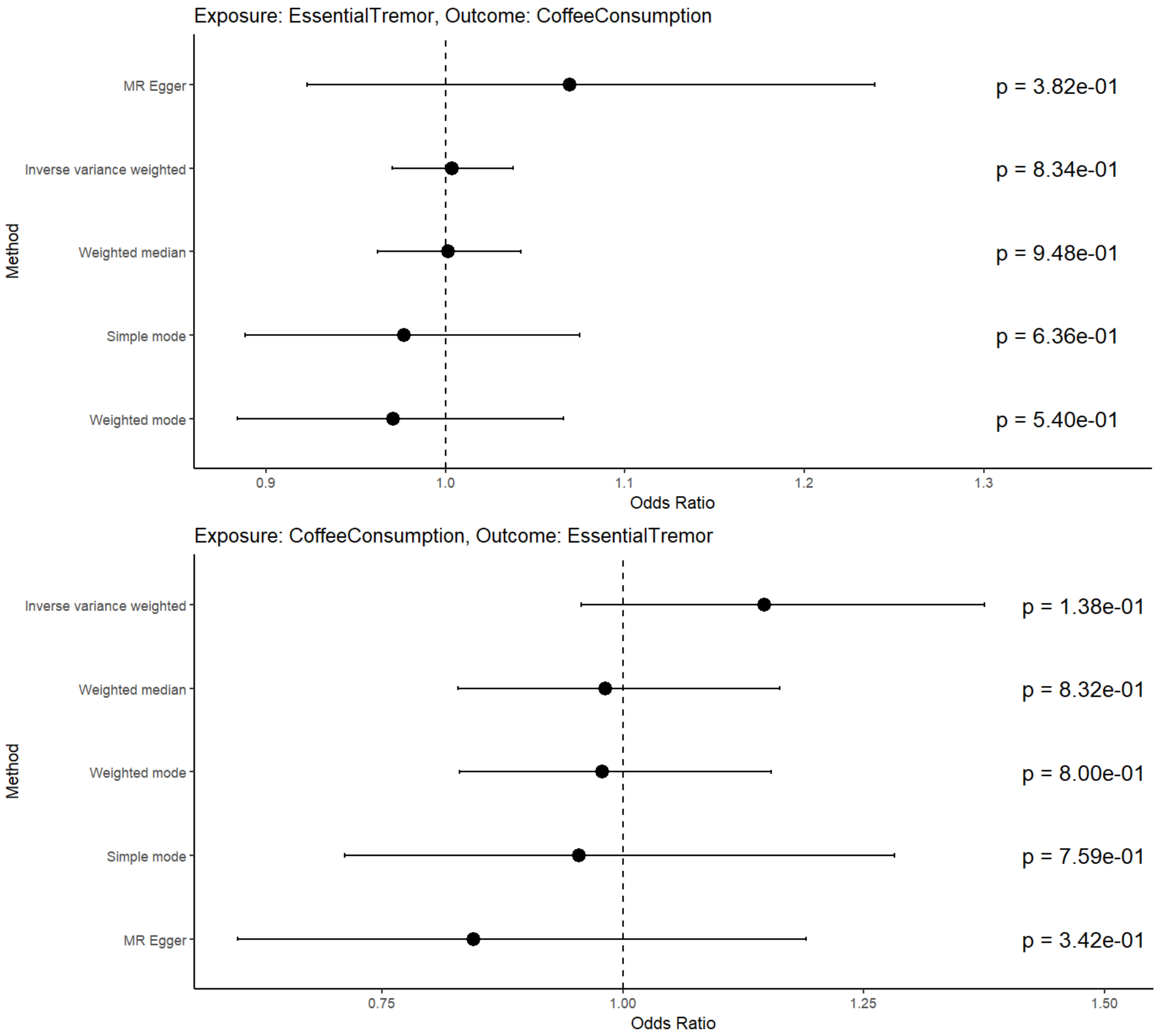

Figure S15: Bidirectional MR results for coffee consumption and essential tremor. The forest plots depict MR analyses results when (A) essential tremor is the exposure with coffee consumption as an outcome, and MR results when (B) coffee consumption is the exposure and essential tremor is the outcome.

For the analysis between coffee consumption and ET, 26 IVs were retained after clumping, harmonizing traits, and Steiger filtering. Methods show no significant effect of ET on coffee consumption, with most methods predicting a weak effect, and contradicting directions (**figure S15**). The LOO plot (**figure S3a**) shows that no IV, when removed, significantly skews the results towards the null hypothesis. Thus, none are disproportionately driving the results. A large Egger intercept is noted (**figure S3b**), suggesting that horizontal pleiotropy may be present. This is also seen in the reverse-direction analysis (**figure S3d**), which further corroborates the presence of horizontal pleiotropy, which is expected to be observed in both directions. However, only one IV was removed during Steiger filtering, and PRESSO did not detect any outliers. In the reverse analysis, conducted with 34 IVs, once again no association is noted, and methods suggest different directions of effect. IV rs1260326 seems to be significantly driving results from LOO analysis

(**figure S3c**), as upon removal of this IV the β-effect drops to 0.07414 from 0.13712. However, no significant result was obtained with or without this IV.

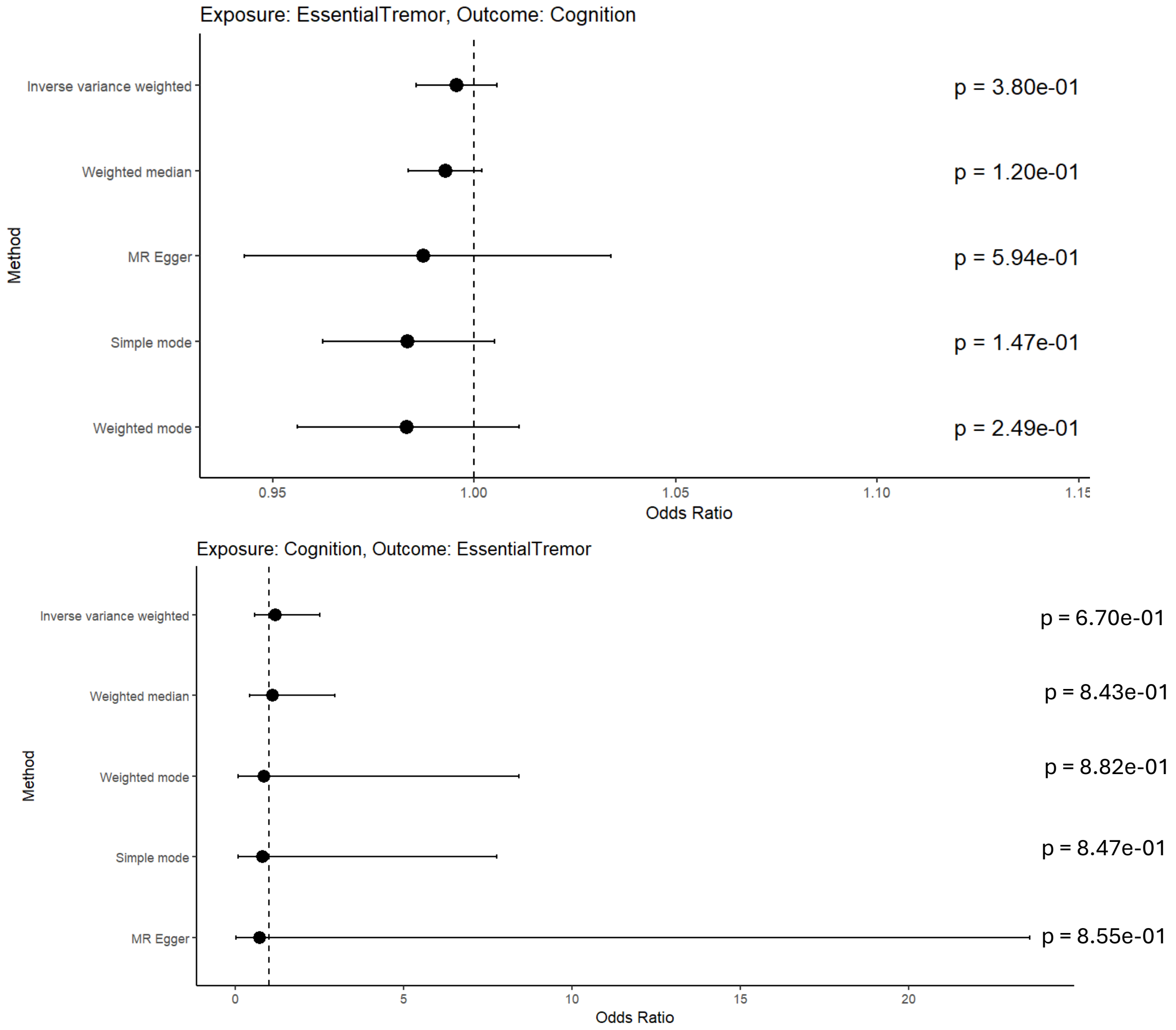

Figure S16: Bidirectional MR results for cognition and essential tremor. The forest plots depict MR analyses results when (A) essential tremor is the exposure with cognition as an outcome, and MR results when (B) cognition is the exposure and essential tremor is the outcome.

21 IVs were retained for the MR analysis with ET as an exposure for cognition. All analytical methods had ORs below 1, however results were all non-significant (**figure S16**). No SNPs were removed from Steiger filtering. PRESSO analysis was not conducted for the cognition MR in either direction, as Steiger filtering and the Egger intercept (see **figure S4b**) showed no evidence for pleiotropic SNPs. From the LOO results (**figure S4a**), rs76187039 was identified as an outlier, as a large deviation (β-effect of -0.00190 with IV removed vs -0.00451 with all IVs) was observed without it. However, as the effect was overall both very weak and insignificant regardless, the sensitivity analysis is irrelevant. The reverse analysis showed a large degree of pleiotropy, with 52 IVs being removed during Steiger filtering. 50 IVs were left to conduct the

analysis. As is seen in **figure S16**, no significant effect is found. From the LOO results (**figure S4c**), it is seen that no single IV was significantly driving results.

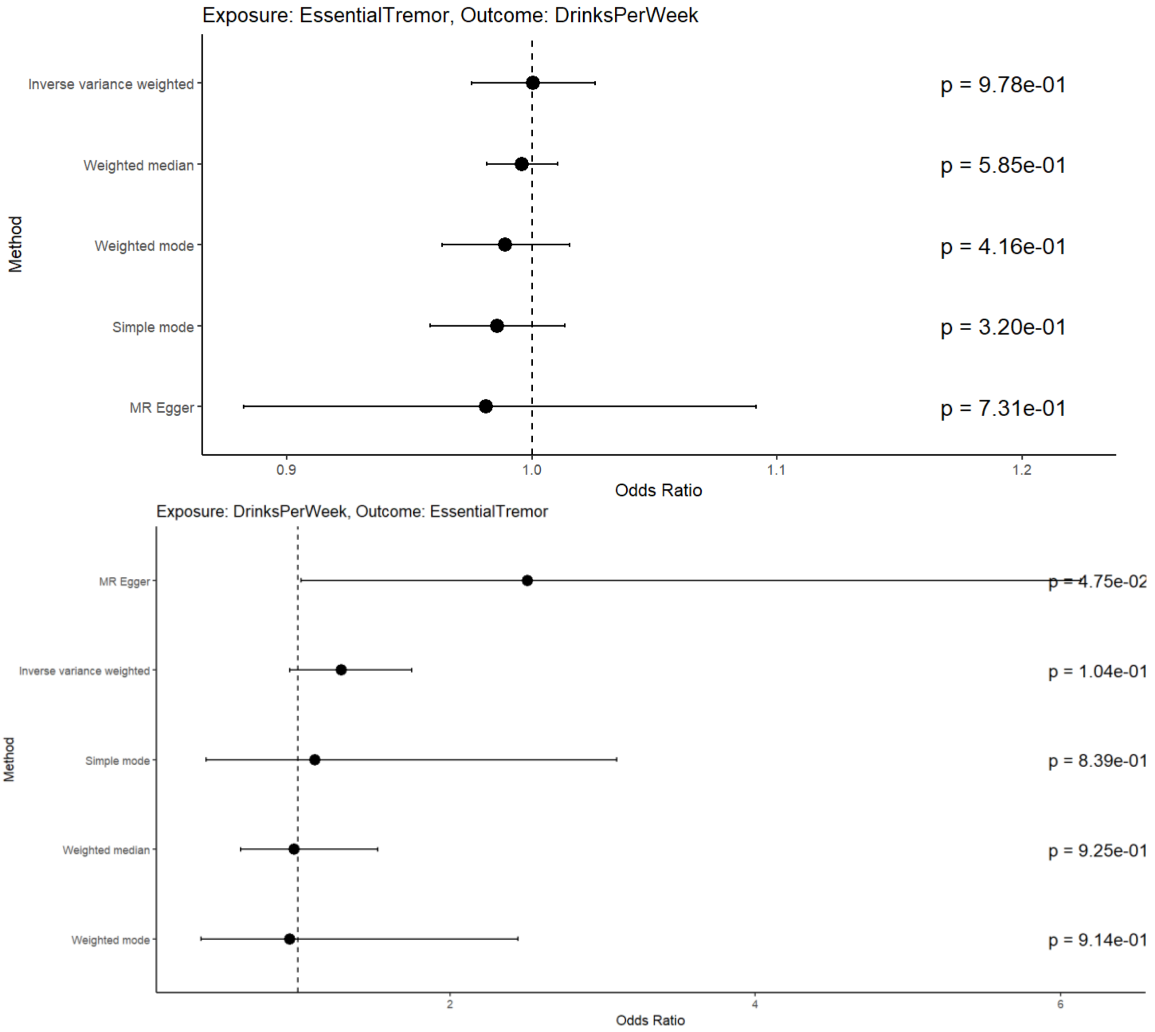

Figure S17: Bidirectional MR results for alcohol intake and essential tremor. The forest plots depict MR analyses results when (A) essential tremor is the exposure with alcohol consumption (drinks per week) as an outcome, and MR results when (B) alcohol consumption is the exposure and essential tremor is the outcome.

The MR analysis between alcohol intake (measured in drinks per week) and ET shows no significant effects, with large p-values across methods and error bars all crossing the null hypothesis, as is seen in **figure S17**. The analysis was conducted with 27 IVs. None were removed after Steiger filtering. As the effect was very small for IVW, the LOO (**figure S5a**) analysis was not very useful, because the effect already tended strongly towards the null. PRESSO analysis did detect outliers, and the recalculated IVW results after correcting for outliers had a p-value of 0.507. The OR however remained the same, being 1.00, suggesting an exactly null effect, however with low confidence. The Egger intercept did not detect any pleiotropy between traits. The global F-statistic for this analysis was 42.923. The reverse analysis, with alcohol intake (measured in drinks

per week) as an exposure for ET, showed an almost significant effect in the Egger regression (p-value 0.0475), however this effect is not robust after multiple-hypothesis correction. Furthermore, this effect is not seen across analytical methods, and other analytical methods suggest an opposite effect. Thus, we cannot consider it significant. The reverse analysis was conducted with 83 IVs, after 21 were Steiger filtered. The global F-statistic was 54.389, suggesting that weak instrument bias was not significant in this analysis. PRESSO analysis did detect outliers, however upon removal of these outliers the p-value significantly increased, jumping from an IVW p-value of 0.108 to a p-value of 0.958. The OR also tended significantly towards the null, with the estimate falling from 1.29 to 1.01, suggesting that the observed effect was driven significantly by pleiotropic SNPs. This suggests a confounding variable may be affecting both alcohol intake and ET. The intercept of the Egger regression however did not suggest this. LOO results (shown in **figure S5c**) showed a dramatic change when rs1260326 was left out, both reducing the magnitude of the effect and changing its direction (-0.06715 with IV removed vs 0.1474 when included). The p-value however remains insignificant (0.714) when the IV is removed. This suggests that the observed association is being strongly driven by this SNP, however in both cases no significant conclusion can be drawn.

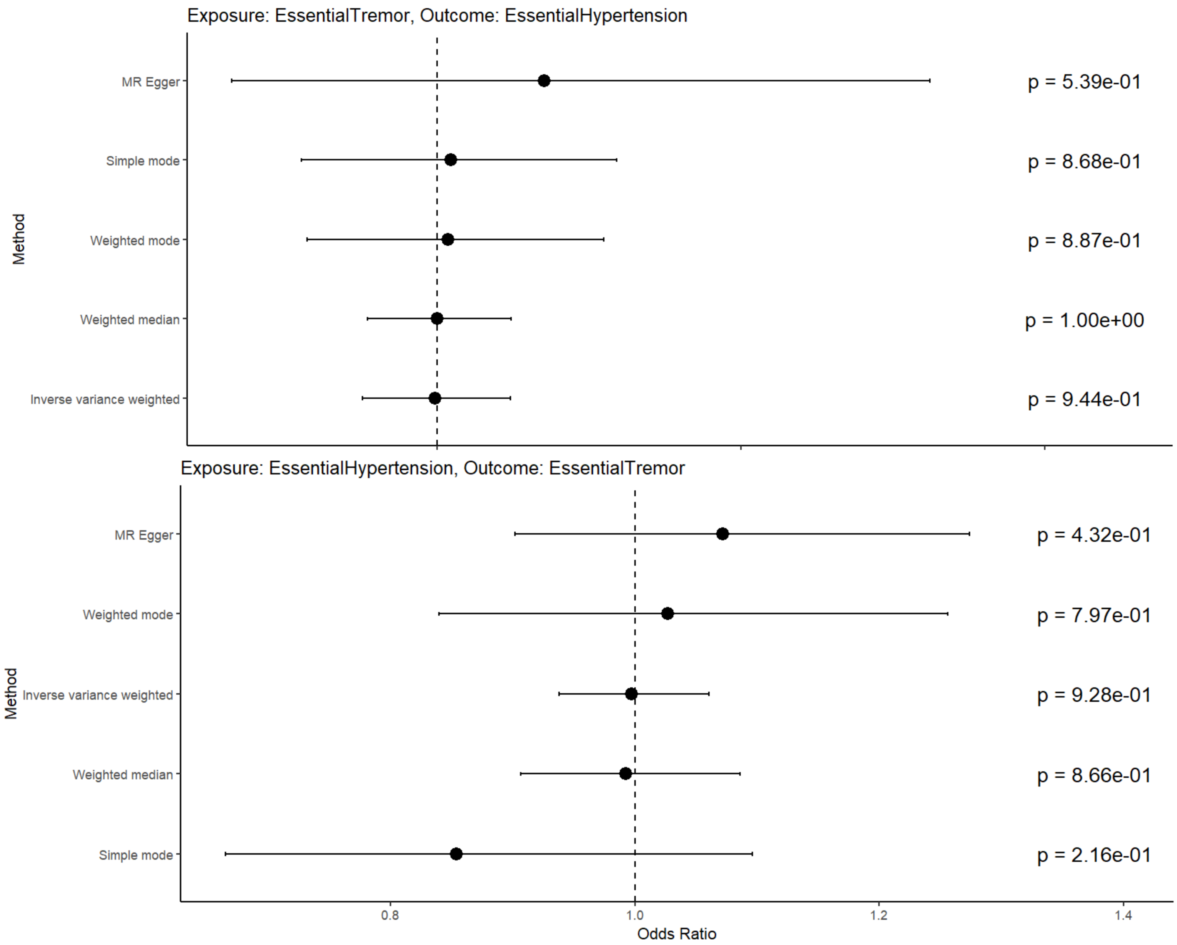

Figure S18: Bidirectional MR results for essential hypertension and essential tremor. The forest plots depict MR analyses results when (A) essential tremor is the exposure with essential hypertension as an outcome, and MR results when (B) essential hypertension is the exposure and essential tremor is the outcome.

For the MR with hypertension as an outcome of ET, shown in **figure S18**, 27 IVs were retained and none were Steiger filtered. The PRESSO analysis detected one outlier, upon removal of which the p-value for the IVW method fell from 0.945 to 0.682, remaining insignificant. The OR shifted very slightly from 1.00 to 1.01. All analyses point towards a null effect, having ORs very close to 1, except the Egger regression. The global F-statistic was 42.817. No single IV seemed to be driving the result from the LOO plot (**figure S6a**). Furthermore, the Egger intercept (see **figure S6b**) was small, so a low level of horizontal pleiotropy is assumed. This is corroborated by the Steiger filtering results, and the low number of outliers detected in PRESSO analysis. For the reverse analysis, 11 SNPs were Steiger filtered, leaving 165 IVs. The high number of Steiger filtered SNPs doesn't necessarily point to a high degree of horizontal pleiotropy, as an unusually high number of SNPs had a significant (p-value $< 5\times10^{-8}$) effect on essential hypertension, so it is likely that some explained more variance in ET. The fact that the PRESSO analysis did not discover any additional outliers and that the LOO plot (**figure S6c**) had an unremarkable distribution further suggests a lack of horizontal pleiotropy; a result found in either direction. It is however worth noting that the LOO analysis is less likely to find a single IV significantly skewing results if the result is calculated from a large number of IVs. The Egger intercept (**figure S6d**) was also unremarkable. The results all point towards a null association, however with low confidence. The global F-statistic was 47.008, confirming that despite the high IV count, weak instrument bias does not affect results.

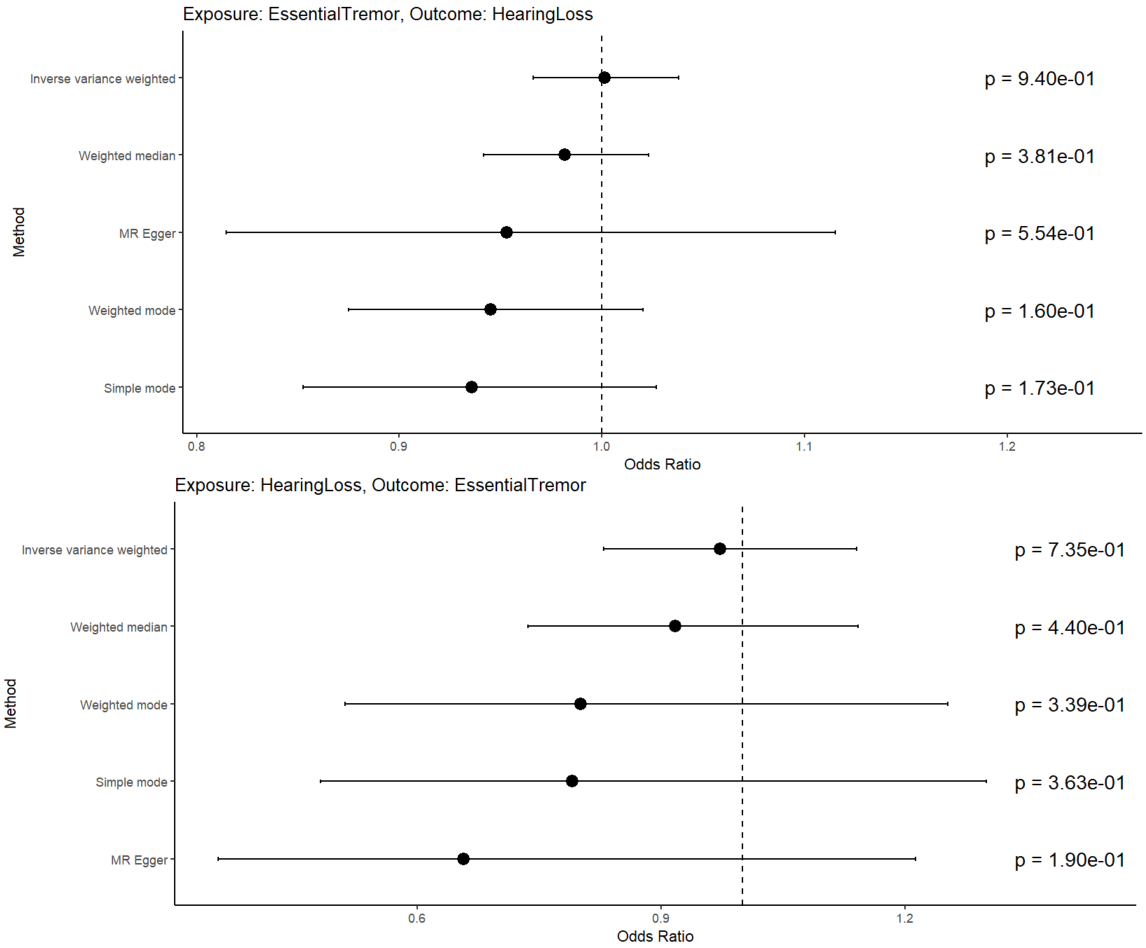

Figure S19: Bidirectional MR results for hearing loss and essential tremor. The forest plots depict MR analyses results when (A) essential tremor is the exposure with hearing loss as an outcome, and MR results when (B) hearing loss is the exposure and essential tremor is the outcome.

27 IVs were found for analyzing the effect of ET as an exposure to hearing loss. None were Steiger filtered. ORs for all methods were close to 1, and all p-values exceeded the threshold for significance (0.0016). PRESSO did not detect any outliers, and the Egger regression intercept was around 0 (**figure S7b**). In the LOO analysis (**figure S7a**), it was seen that the direction of the effect was changed when removing some IVs. However, as the IVW effect prediction was already small (β-effect of 0.0013), this is not surprising. The global F-statistic was 42.817. Horizontal pleiotropy is not likely to be driving results, as the Egger intercept is small, and both Steiger filtering and PRESSO failed to detect pleiotropic SNPs. Thus, results suggest a null association; however, they don't do so conclusively. In the reverse analysis, with ET as an outcome of hearing loss, 30 IVs were found, after one was discarded during Steiger filtering. All results showed a weakly negative effect; however, none were significant, as seen in **figure S19**. Once again, the LOO plot (**figure S7c**) showed a change in the direction of the effect after removing certain IVs, however due to the small effect size (β-effect of 0.00561 with all IVs) this is not remarkable. From the regression plot (**figure S7d**), one SNP with a particularly large effect on hearing loss can be seen. The Egger

regression also has a higher intercept than other methods. This is likely due to the aforementioned SNP, as it significantly skews towards a negative result. This can be observed from the LOO results, where removing that SNP increases the effect nearly ten-fold, from 0.00561 with all IVs to 0.0464 without it, and furthermore the p-value decreases dramatically from 0.947 to 0.583. Removing this SNP would be overfitting data, as it has a strong effect on the exposure trait, and was not removed by Steiger filtering or PRESSO analysis.

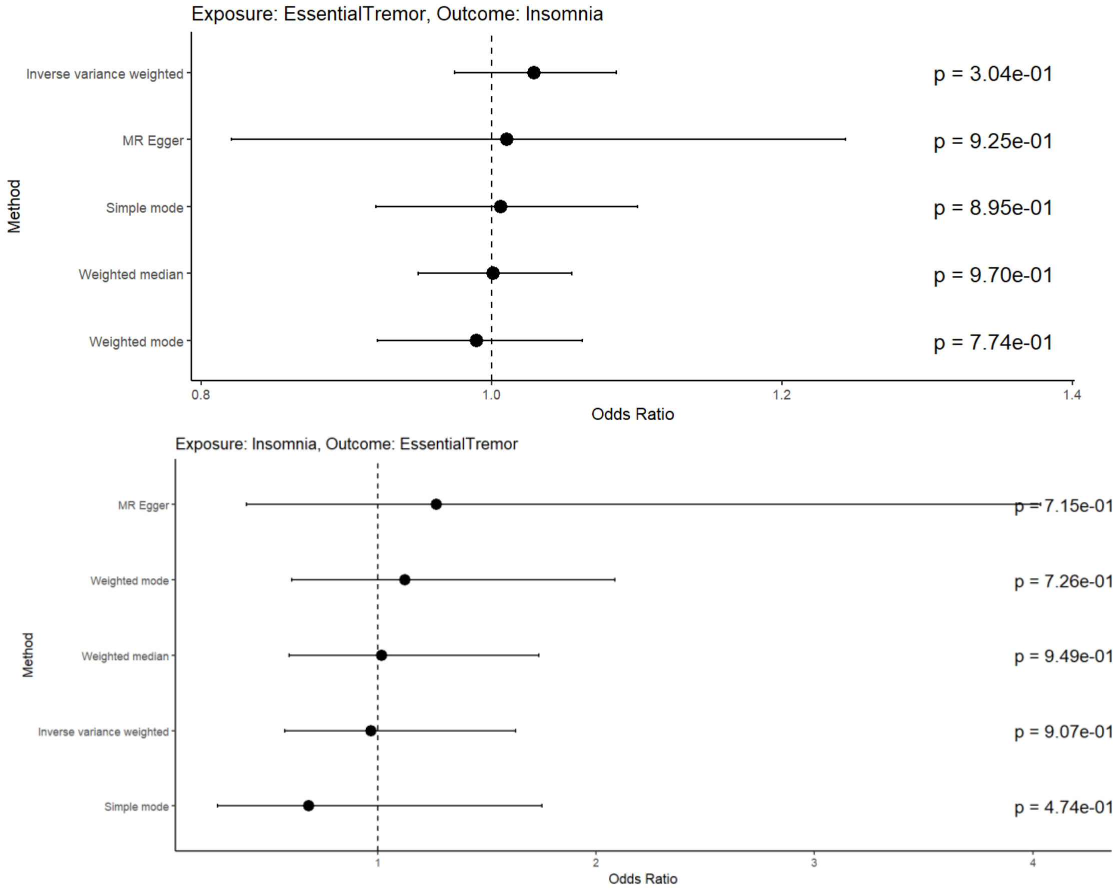

Figure S20: Bidirectional MR results for insomnia and essential tremor. The forest plots depict MR analyses results when (A) essential tremor is the exposure with insomnia as an outcome, and MR results when (B) insomnia is the exposure and essential tremor is the outcome.

Only 12 IVs were retained for the MR with ET as an exposure for insomnia. No IVs were Steiger filtered therefore the low number of IVs is likely due to the insomnia GWAS [35] lacking overlapping SNPs with the ET GWAS. [8] The F-statistic was 43.029, so IVs were sufficiently powerful. As seen in **figure S20**, no results were significant. Furthermore, for the IVW analysis (which had the largest OR), PRESSO analysis detected 1 outlier, upon removal of which the OR was reduced from 1.029 to 1.006, with a p-value change from 0.326 to 0.784. The Egger intercept (**figure S8b**) was small, so pleiotropy may not be present. As the OR already tended towards the null, LOO analysis (**figure S8a**) did not identify any IVs that should be discarded. With ET as an outcome of insomnia, only 7 candidate IVs were found, of which 2 were eliminated during Steiger filtering (leaving only 5 IVs for the analysis). PRESSO did not identify any additional outliers.

The low number of IVs increases the uncertainty of the analysis, as is seen from the high p-values in **figure S20**. As there was such a small number of IVs, the LOO analysis (**figure S8c**) is more volatile, as each IV makes up a large portion of the analysis. Therefore, it is not a useful metric in this case. The global F-statistic for the analysis was 44.871, indicating a low level of weak instrument bias. Nonetheless, due to the low confidence and few SNPs left for the analysis, no significant association is found in either direction.

Figure S21: Bidirectional MR results for LDL levels and essential tremor. The forest plots depict MR analyses results when (A) essential tremor is the exposure with low-density lipoprotein (LDL) as an outcome, and MR results when (B) LDL is the exposure and essential tremor is the outcome.

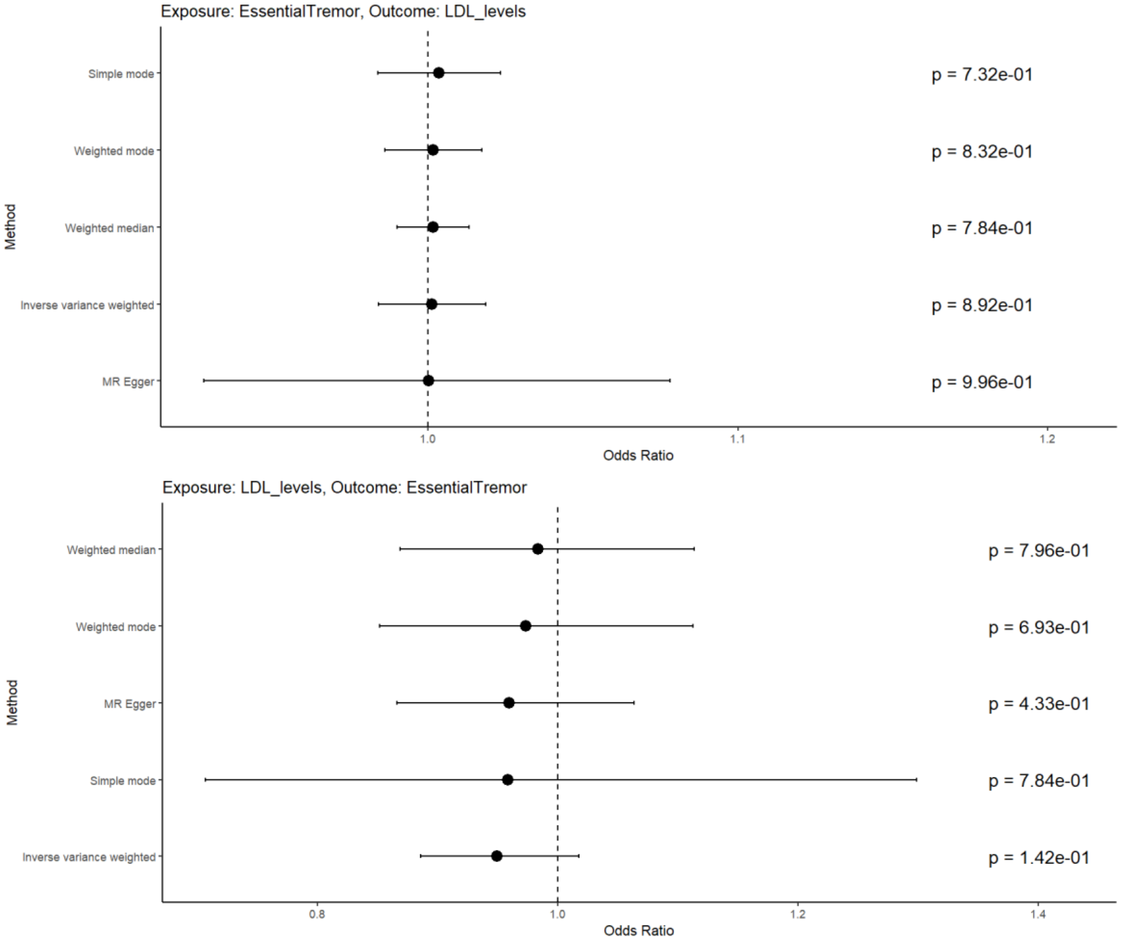

For the MR with ET as an exposure for LDL levels, 25 IVs were retained after 2 were removed from Steiger filtering. The global F-statistic was 43.598. From the LOO plot (**figure S9a**), rs780093 is seen to significantly skew results, however this SNP was already discarded from the analysis via Steiger filtering. From **figure S20** we note that all ORs are small and have high p-values, so no conclusion can be drawn. The Egger intercept (**figure S9b**) suggests that some horizontal pleiotropy may be present. PRESSO also suggests this, as 4 outliers were removed. Despite this, results upon outlier removal did not change much (prior to outlier removal: IVW OR

1.001, p-value 0.893, after outlier removal: IVW OR 1.000, p-value 0.869). For the reverse analysis (with LDL levels as an exposure for ET), 83 IVs were Steiger filtered, leaving 438 for the analysis. The large number of IVs is due to the high number of SNPs strongly correlated to LDL levels in the LDL GWAS. [36] Because of the large number of IVs, the LOO plot is excluded from **figure S9**, as it is too dense to be of use. No IV was found to significantly drive results, which is expected given the high number of IVs used for the analysis. The Egger intercept (**figure S9c**) is small, suggesting minimal horizontal pleiotropy. However, the large proportion of SNPs removed due to Steiger filtering contradicts this. As the regression is calculated after the removal of those SNPs, horizontal pleiotropy is still likely. The global F-statistic was exceptionally high, being 266.168. No effect can be elucidated despite the high number and power of SNPs, results are all non-significant (shown in **figure S20**).

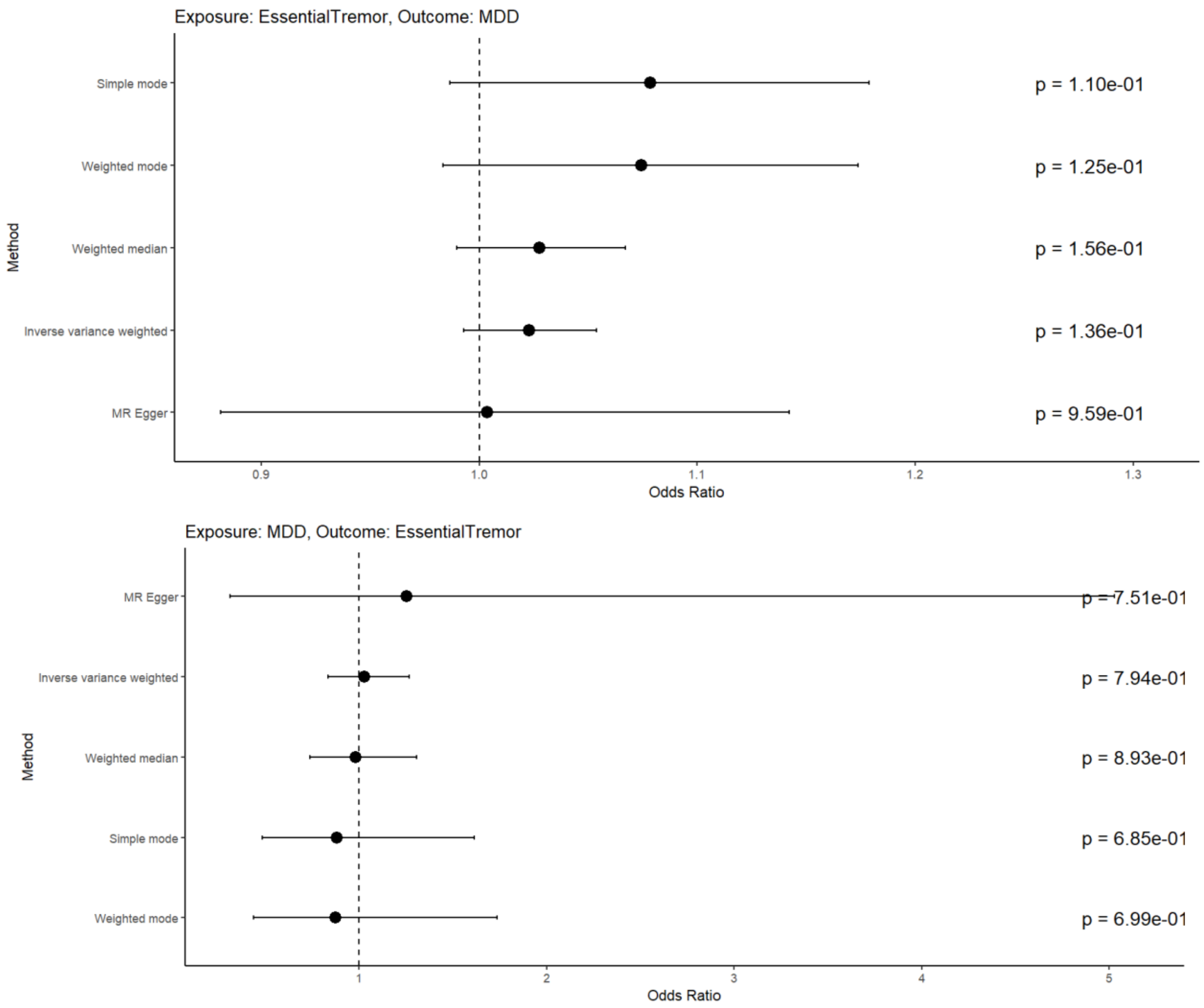

Figure S21: Bidirectional MR results for MDD and essential tremor. The forest plots depict MR analyses results when (A) essential tremor is the exposure with major depression disorder (MDD) as an outcome, and MR results when (B) MDD is the exposure and essential tremor is the outcome.

MR analysis with ET as an exposure for MDD was conducted with 24 IVs. None were Steiger filtered. The global F-statistic was 42.455, suggesting weak instrument bias was minimal, if present at all. Though all analytic methods generally point to a positive effect of ET on MDD, no result was significant (**figure S21**). PRESSO did not detect any outliers for the analysis. From the LOO plot (**figure S10a**), no single IV seems to be driving results. The Egger intercept (**figure S10b**) was near zero. The reverse analysis (MDD as an exposure for ET) had 45 candidate IVs, of which 13 were Steiger filtered, leaving 32 for the analysis. The global F-statistic for these SNPs was 40.097. The high number of filtered SNPs suggest horizontal pleiotropy is likely. The Egger regression (**figure S10d**) is small, however once again this is after the removal of pleiotropic SNPs. The LOO plot (**figure S10c**) does not suggest any outlier IVs. Interestingly, in the analysis prior to Steiger filtering, results appeared to be significant. The estimated OR by IVW calculations was 1.419, with a p-value of 0.00639. However, this significance was lost after removal of the pleiotropic SNPs, which strongly suggests pleiotropy between the two traits. While neither directional nor causal associations can be drawn from this result (see **figure S21**), it does give evidence for horizontal pleiotropy that merits further study.

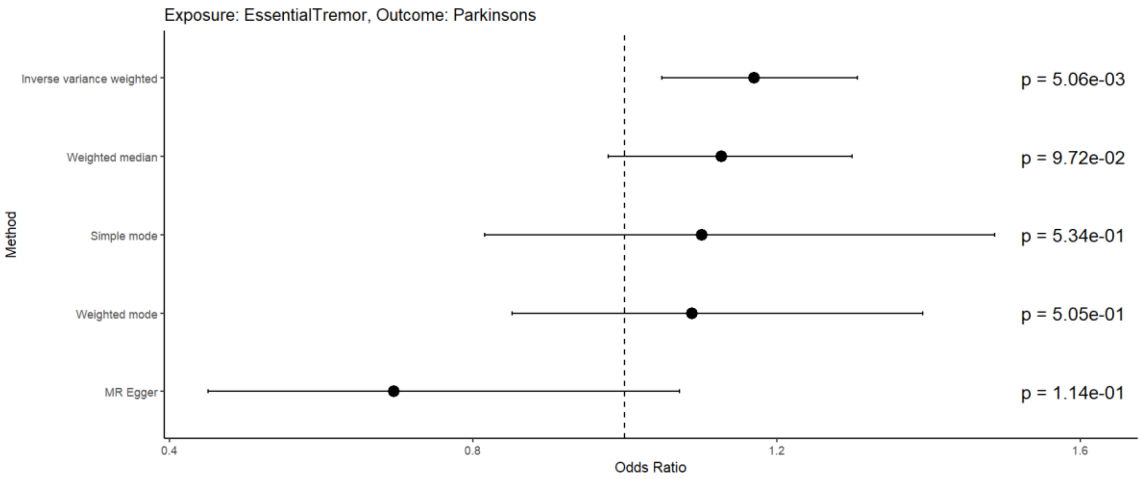

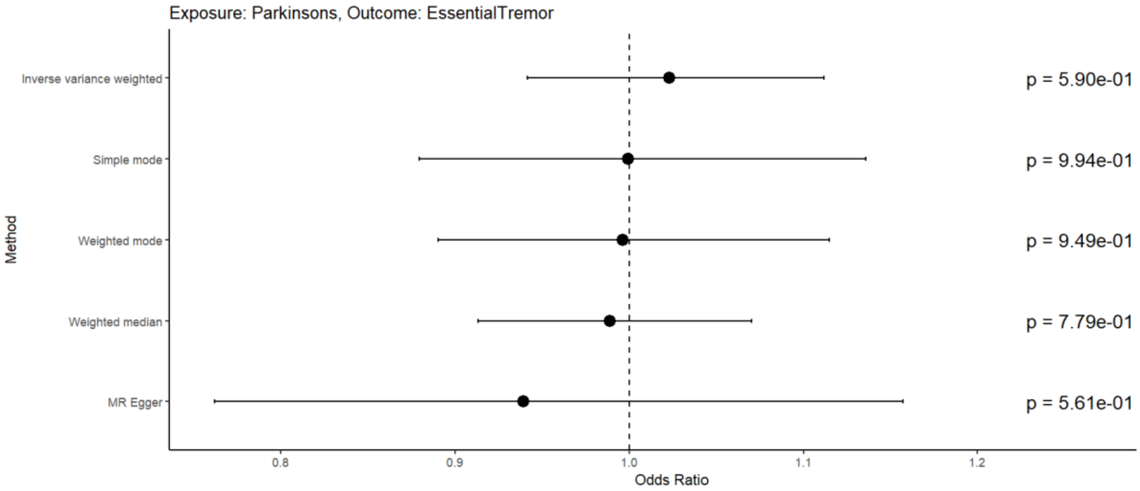

Figure S22: Bidirectional MR results for Parkinson's disease and essential tremor. The forest plots depict MR analyses results when (A) essential tremor is the exposure with Parkinson's disease (PD) as an outcome, and MR results when (B) PD is the exposure and essential tremor is the outcome.

With ET as an exposure for Parkinson's disorder (PD), one IV was Steiger filtered, leaving 25 for the analysis. The global F-statistic was 43.589. From **figure S22** we see most methods point towards a positive effect of ET on PD, and in fact a significant p-value is seen in the IVW calculation of effect (however this association does not survive multiple hypothesis correction). The Egger regression shows a strong (but insignificant) effect in the opposite direction, however. Upon closer inspection (see **figure S11b**), a striking y-intercept is seen in the Egger test, suggesting a large degree of horizontal pleiotropy between ET and PD. This is also reported in literature. [16] Despite the obvious presence of pleiotropy, PRESSO failed to detect outliers, perhaps due to how widespread the pleiotropy is. LOO analysis (**figure S11a**) also fails to detect this, suggesting a multitude of pleiotropic SNPs. Thus, in addition to the fact that the effect observed in IVW analysis is not robust across analytic methods, we also can't draw a conclusion as the 3rd assumption of MR (that there is no pathway between the IVs and the outcome independent of the exposure) is likely violated. [25] The reverse-direction analysis (with ET as an outcome of PD) was conducted with 23 IVs. No IVs were removed during Steiger filtering, however PRESSO removed 3 outliers. Results were insignificant even with outliers and remained so after their removal (see **figure S22**). The recalculated IVW OR was 1.005, with a p-value of 0.867. The Egger intercept was not as dramatic in the reverse analysis, as seen in **figure S11d**. The global F-statistic for the reverse direction was 54.752. LOO results (**figure S11c**) suggest rs10847864 may be the influence driving results (β-effect of 0.004514 when it is removed vs 0.02290 when it's present). This SNP was also detected by PRESSO. Therefore, horizontal pleiotropy is observed in either direction.

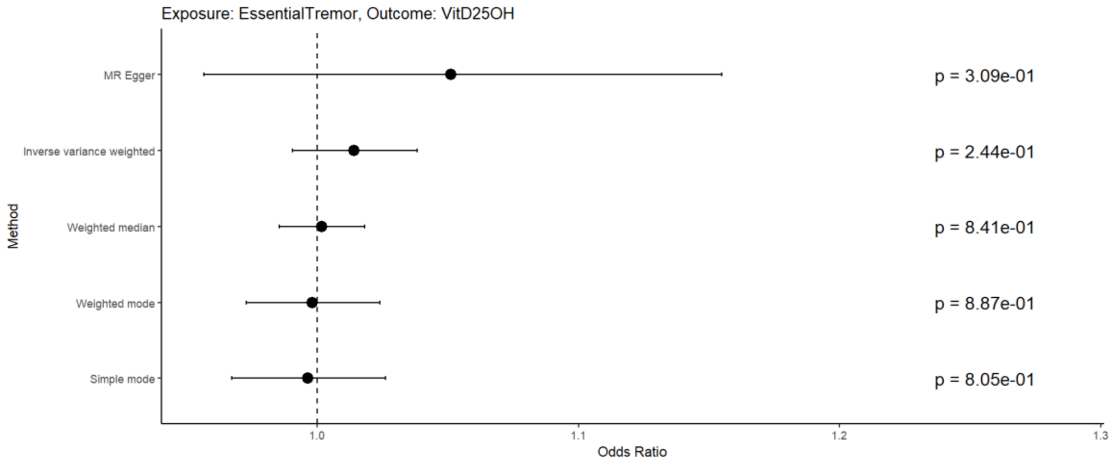

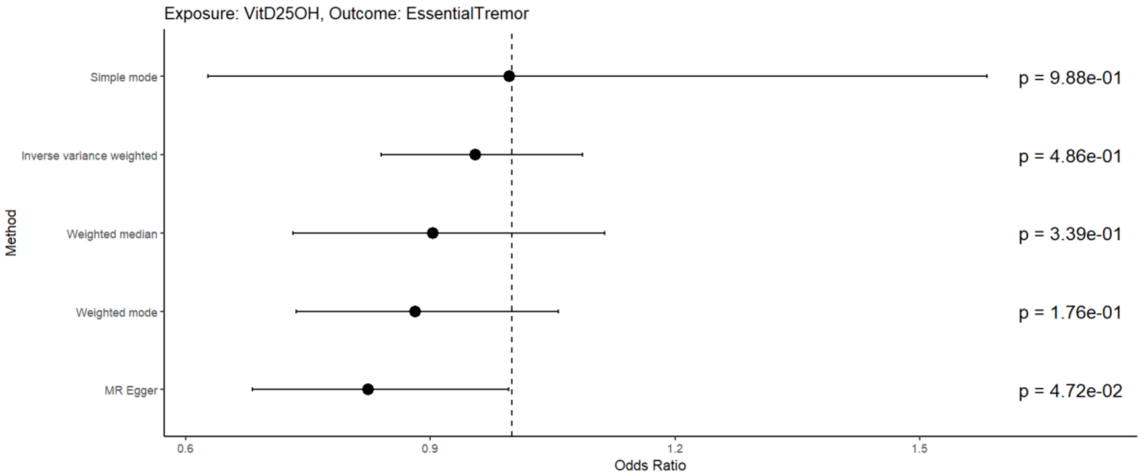

Figure S23: Bidirectional MR results for 25-OH Vit. D levels and essential tremor. The forest plots depict MR analyses results when (A) essential tremor is the exposure with 25-OH Vitamin D levels as an outcome, and MR results when (B) 25-OH Vitamin D levels is the exposure and essential tremor is the outcome.

27 IVs were retained in the MR with ET as an exposure to serum 25-OH Vitamin D levels. None were Steiger filtered. The global F-statistic was 42.923. All ORs were near 1, as seen in **figure S23**, except the Egger regression which predicted a slightly greater effect, however with large error. P-values indicate that no association can be inferred. The LOO plot (**figure S12a**) suggests that rs780093 may be significantly driving results, as the β-effect drops from 0.0140 when it is included to 0.00664 when it is excluded. PRESSO results suggest the IV should be excluded as well, however results remain insignificant with or without it. For the reverse analysis (vitamin D levels as an exposure for ET), 12 IVs were Steiger filtered, leaving 122 for analysis. From **figure S22**, although we see that the Egger regression proposes a moderate effect, which would suggest that low 25-OH vitamin D levels confer risk for ET, the p-value is ultimately insignificant. Additionally, other analytic methods do not support such a conclusion. The global F-statistic was high, being 158.365. LOO analysis (**figure S12c**) identifies rs1260326 as significantly driving results (β-effect -0.00910 without vs 0.02687 with), however it was already removed from the primary analysis during Steiger filtering.

For the brain phenotypes, IV retention was not sufficient to conduct MR with brain volume measures as exposures for ET. Only one IV was found for both left and right cerebellar grey matter volumes. Only three IVs were present for left cerebellar white matter, and two for right cerebellar white matter. Thus, the MR results are not reported for brain phenotypes, as the analysis could not be reliably conducted bidirectionally.

Notably, rs780093 is often detected by LOO analysis of ET-as-exposure MRs because it has a strong p-value (5.51×10^{-20}) in the ET GWAS, [8] thus has a large weight in IVW analysis (the method used for the LOO analysis).

Once again, no causal and directional associations could be made between ET and other traits via MR. However, interesting horizontal pleiotropy was noted in many analyses, which may partially be obscuring MR, which seeks to regress out genetic correlations.

Pleiotropic SNP Analysis: Pathway Enrichment

After identifying pleiotropic SNPs between the pairings of studied traits with ET, SNPs were mapped to the genes they reside in, and then pathways enrichment analyses were conducted for all pairs. Results of the top ten most significantly enriched terms per paired trait analysis with ET are shown below.

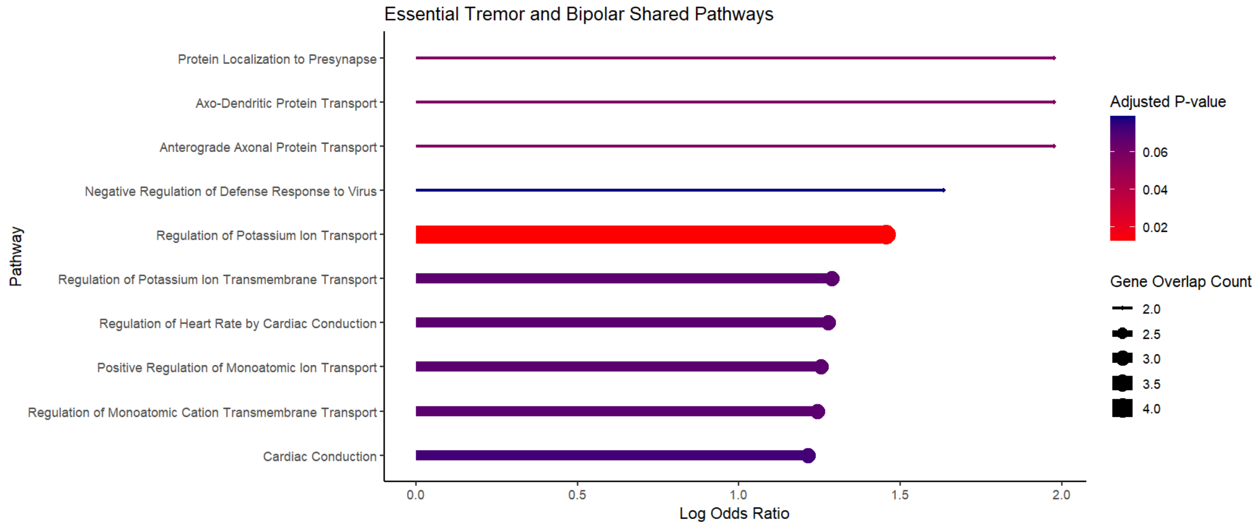

Figure S24: Pathways with shared association between ET and bipolar disorder. Geneset enrichment analyses were done using the genes that pleiotropic SNPs resided in between ET and bipolar disorder. Enrichment terms were derived from Enrichr "GO Biological Terms 2025". The ten most significant terms are presented.

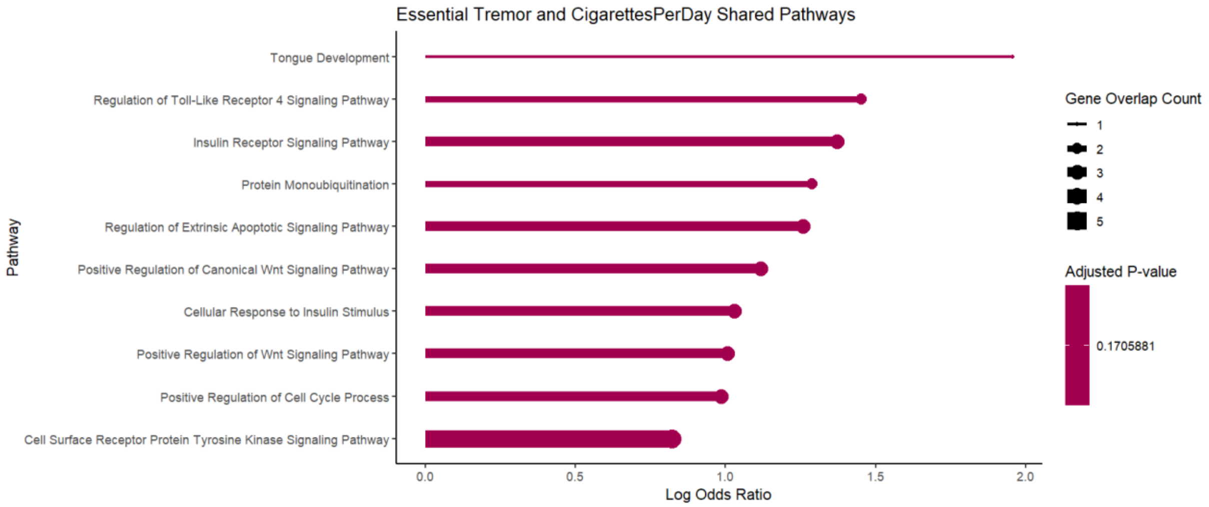

Figure S25: Pathways with shared association between ET and smoking. Geneset enrichment analyses were done using the genes that pleiotropic SNPs resided in between ET and number of cigarettes smoked. Enrichment terms were derived from Enrichr “GO Biological Terms 2025”. The ten most significant terms are presented.

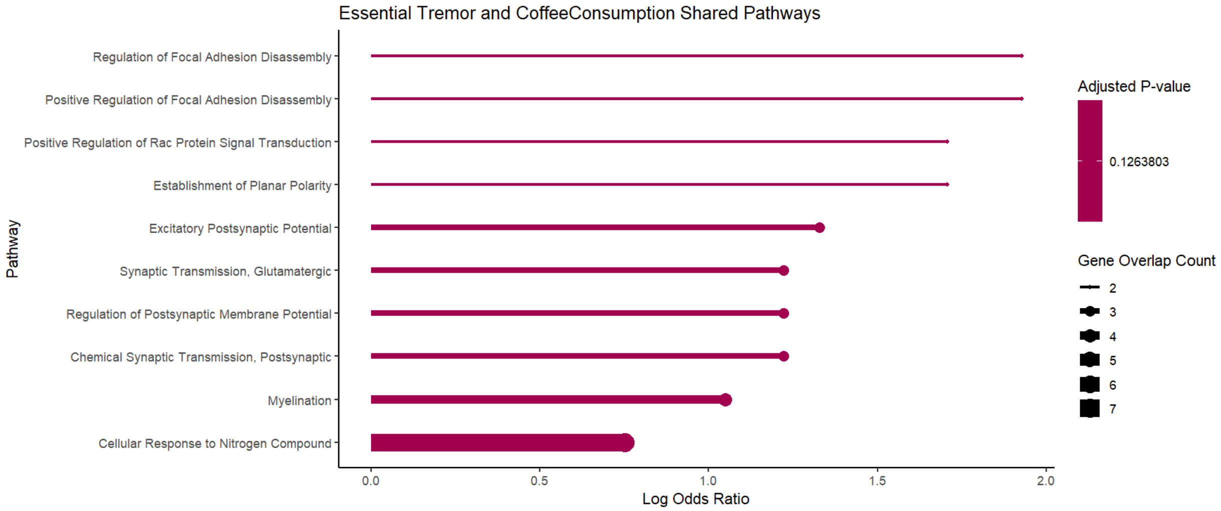

Figure S26: Pathways with shared association between ET and coffee consumption. Geneset enrichment analyses were done using the genes that pleiotropic SNPs resided in between ET and coffee consumption. Enrichment terms were derived from Enrichr “GO Biological Terms 2025”. The ten most significant terms are presented.

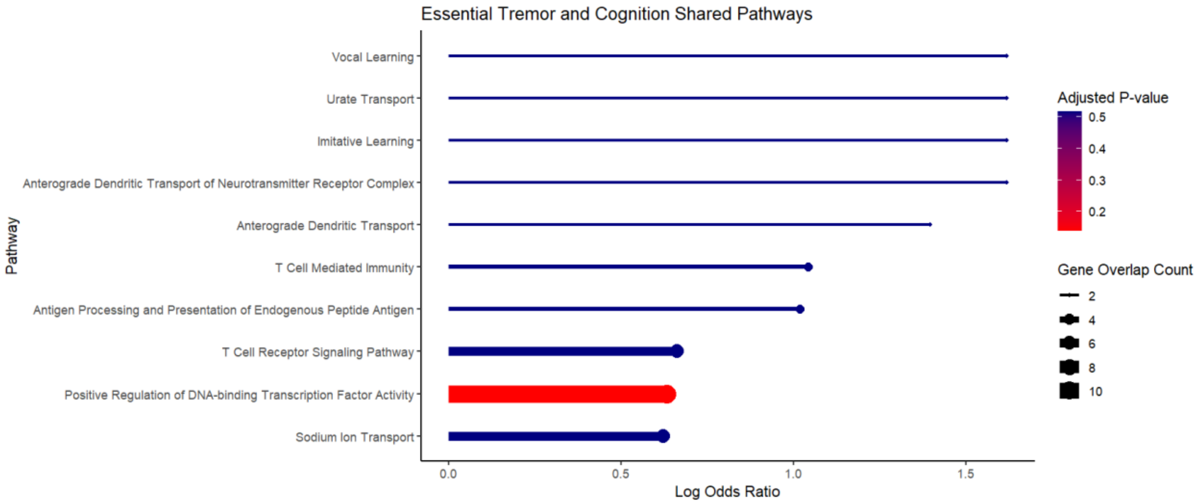

Figure S27: Pathways with shared association between ET and cognition. Geneset enrichment analyses were done using the genes that pleiotropic SNPs resided in between ET and cognition. Enrichment terms were derived from Enrichr "GO Biological Terms 2025". The ten most significant terms are presented.

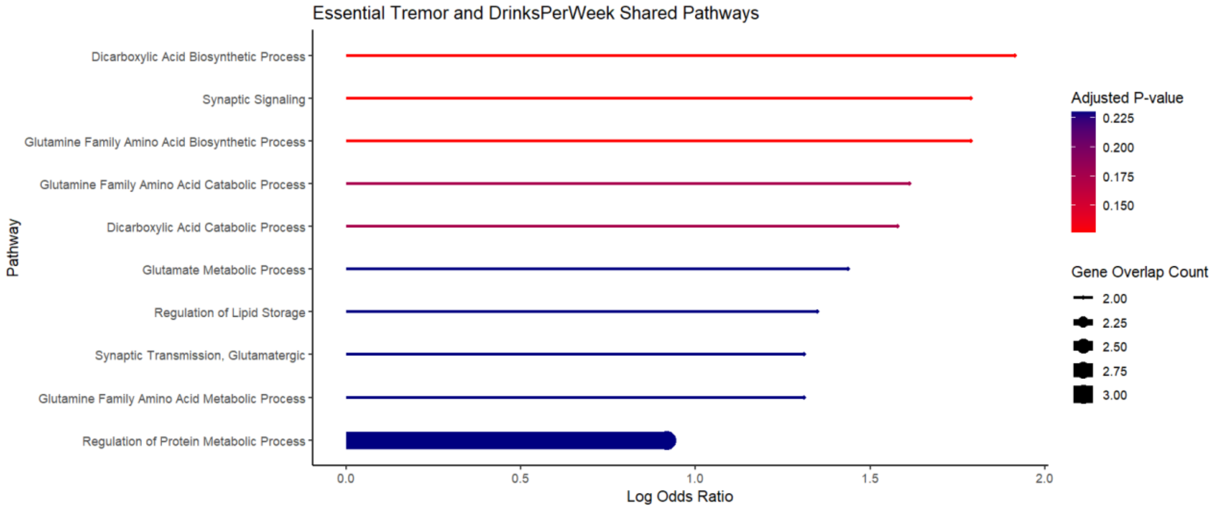

Figure S28: Pathways with shared association between ET and alcohol intake. Geneset enrichment analyses were done using the genes that pleiotropic SNPs resided in between ET and alcohol intake. Enrichment terms were derived from Enrichr "GO Biological Terms 2025". The ten most significant terms are presented.

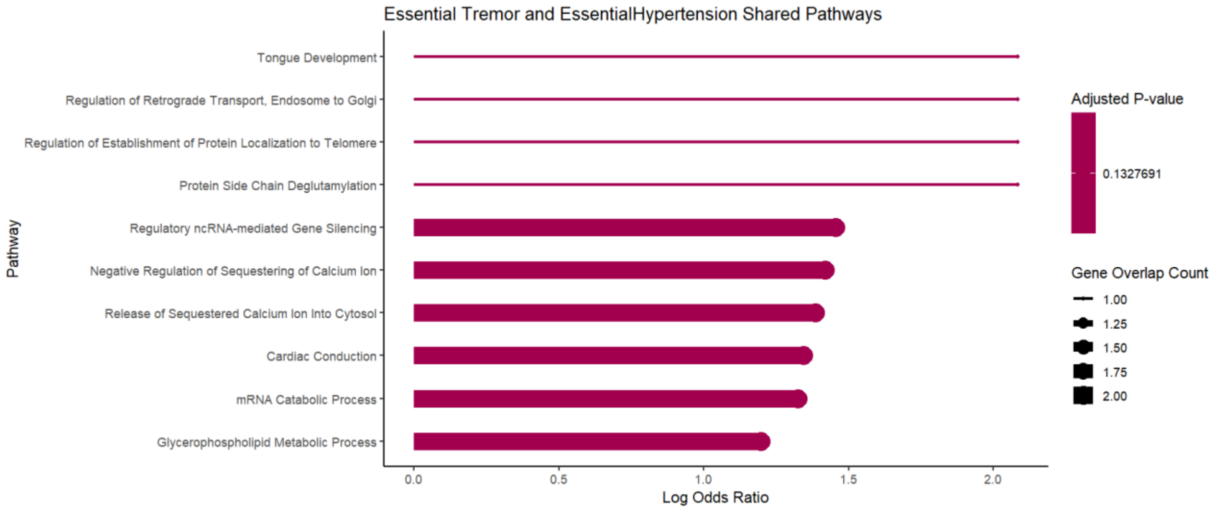

Figure S29: Pathways with shared associations between ET and essential hypertension. Geneset enrichment analyses were done using the genes that pleiotropic SNPs resided in between ET and essential hypertension. Enrichment terms were derived from Enrichr "GO Biological Terms 2025". The ten most significant terms are presented.

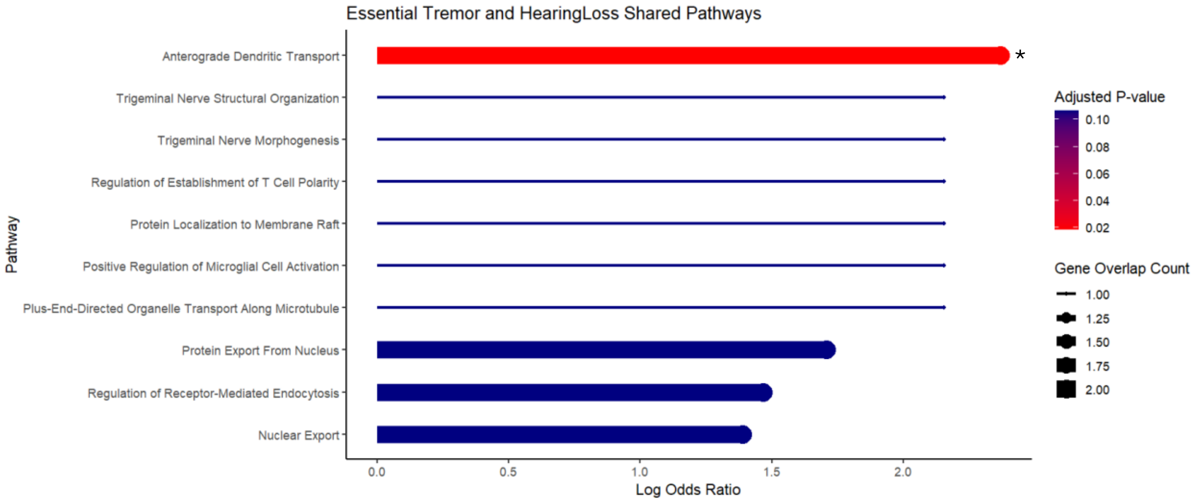

Figure S30: Pathways with shared associations between ET and hearing loss. Geneset enrichment analyses were done using the genes that pleiotropic SNPs resided in between ET and hearing loss. Enrichment terms were derived from Enrichr "GO Biological Terms 2025". The ten most significant terms are presented.

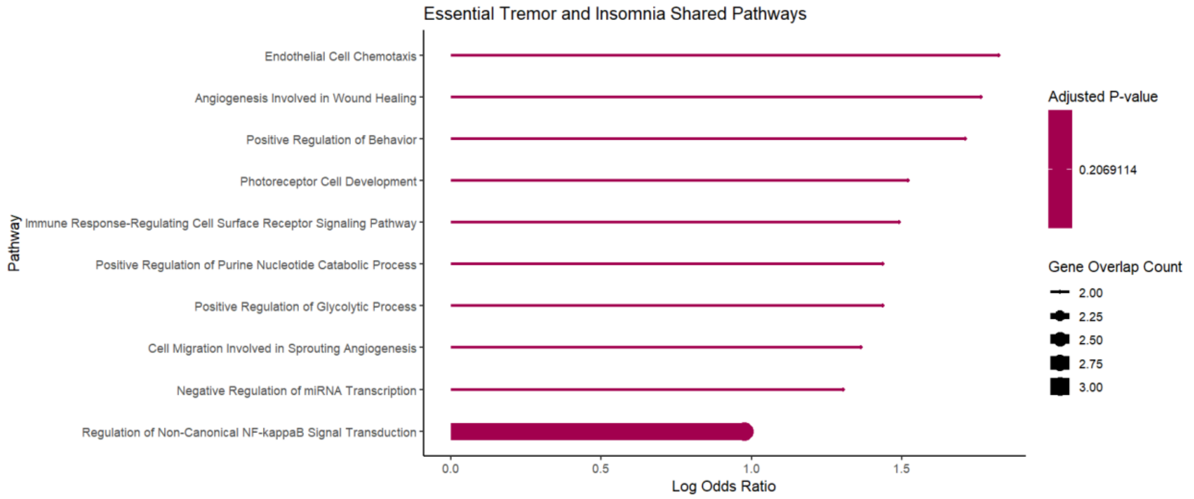

Figure S31: Pathways with shared associations between ET and insomnia. Geneset enrichment analyses were done using the genes that pleiotropic SNPs resided in between ET and insomnia. Enrichment terms were derived from Enrichr "GO Biological Terms 2025". The ten most significant terms are presented.

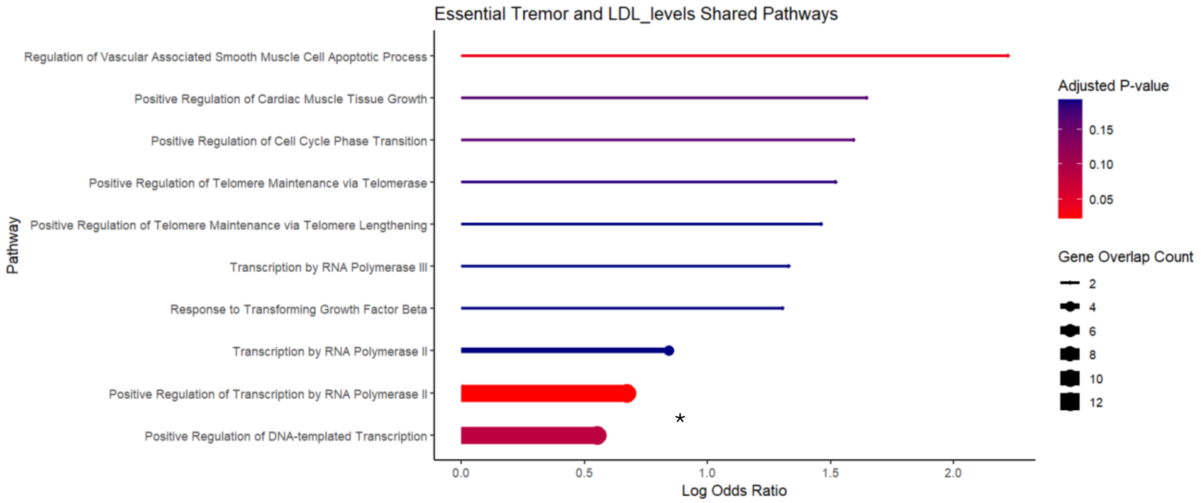

Figure S31: Pathways with shared associations between ET and LDL levels. Geneset enrichment analyses were done using the genes that pleiotropic SNPs resided in between ET and LDL levels. Enrichment terms were derived from Enrichr "GO Biological Terms 2025". The ten most significant terms are presented.

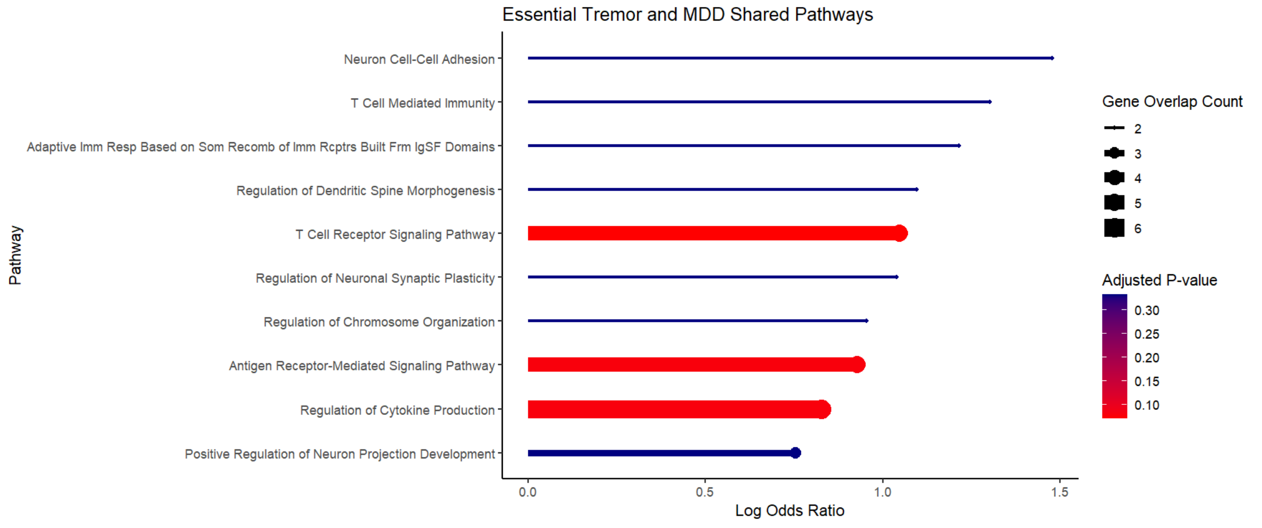

Figure S32: Pathways with shared associations between ET and MDD. Geneset enrichment analyses were done using the genes that pleiotropic SNPs resided in between ET and MDD. Enrichment terms were derived from Enrichr "GO Biological Terms 2025". The ten most significant terms are presented.

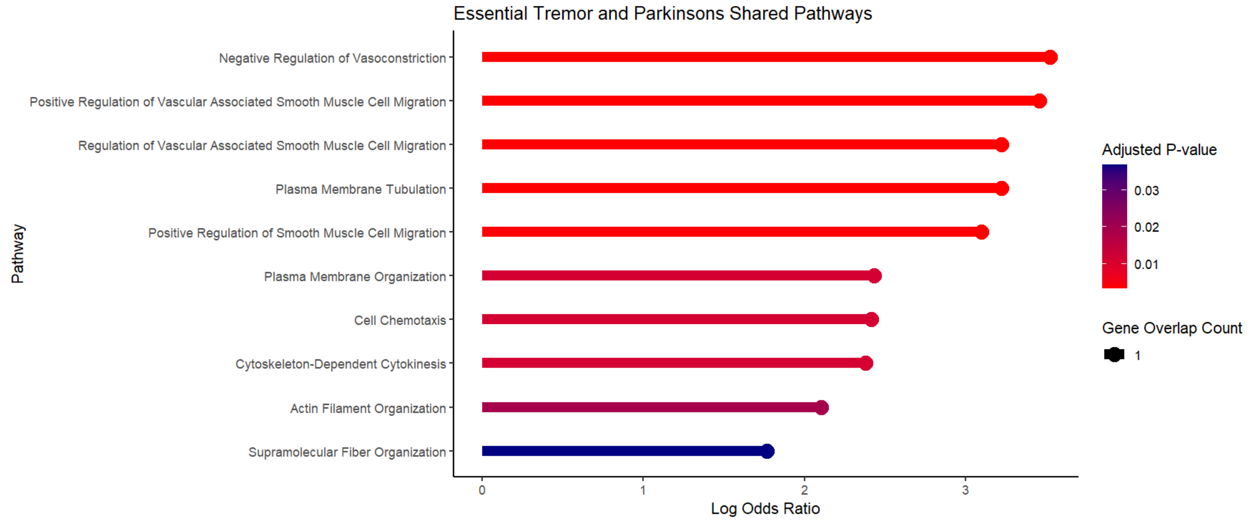

Figure S33: Pathways with shared associations between ET and Parkinson's disease. Geneset enrichment analyses were done using the genes that pleiotropic SNPs resided in between ET and Parkinson's disease. Enrichment terms were derived from Enrichr "GO Biological Terms 2025". The ten most significant terms are presented.

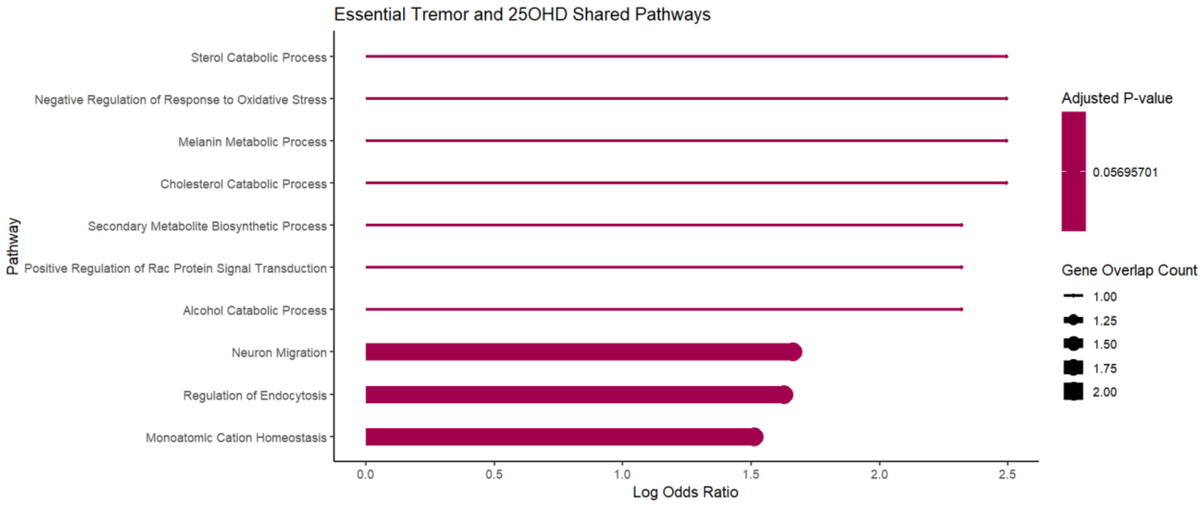

Figure S34: Pathways with shared associations between ET and 25-OH Vitamin D levels. Geneset enrichment analyses were done using the genes that pleiotropic SNPs resided in between ET and 25-OH Vitamin D. Enrichment terms were derived from Enrichr "GO Biological Terms 2025". The ten most significant terms are presented.

Genomic Structural Equation Modeling: linkage disequilibrium trait correlation matrix

To understand how related traits were with one another and ET and to aid in partitioning traits into latent variables for genomic structural equation modeling, an LDSC genetic correlation matrix was computed for all traits shown below.

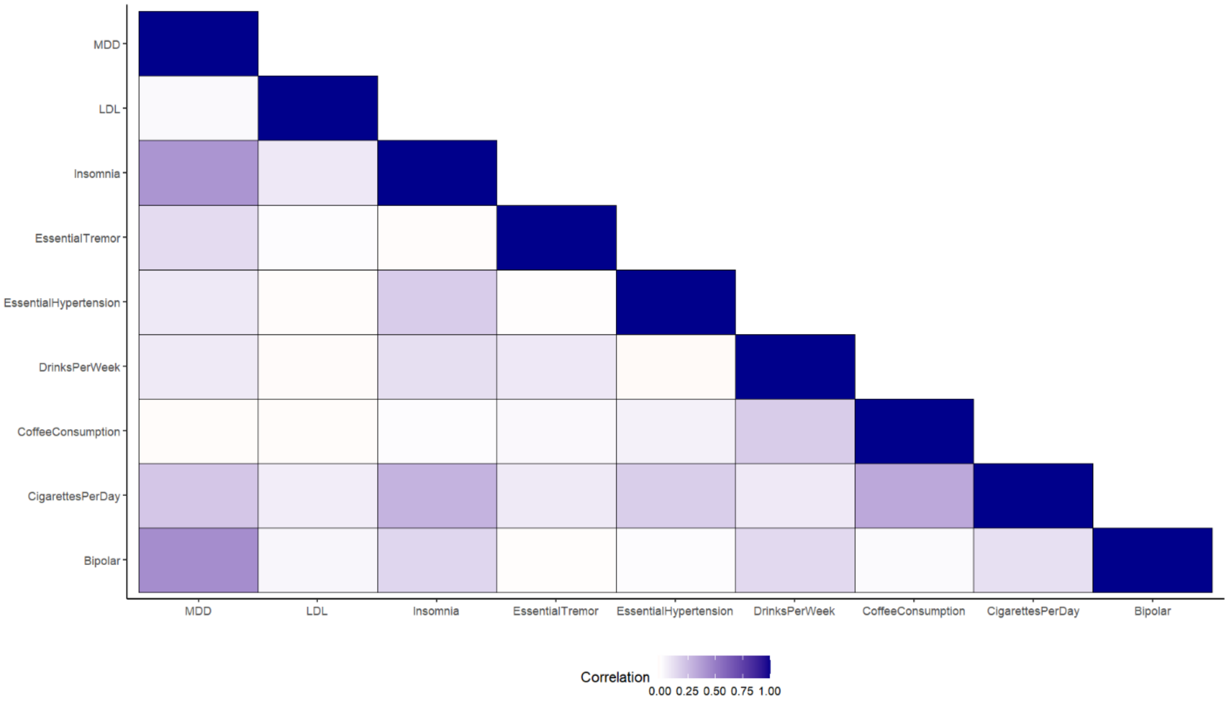

Figure S35: LD Correlation matrix of ET-associated phenotypes. In the covariance matrix, stronger linkage disequilibrium correlations are represented by darker colors. ET is seen to have little genetic correlation with other traits, except MDD (which has been previously reported in the GWAS used in our experiment). Other well-known associations are also noticed, like between bipolar disorder and MDD. From this covariance matrix, we observe that bipolar disorder, MDD, and insomnia have shared genetic architecture, therefore informing a candidate latent variable. Alcohol intake (drinks per week) and coffee consumption also share some correlation. A similar observation was made with LDL levels, smoking, and hypertension. Smoking and coffee consumption are also correlated.
